# Supplementary material for: Motivators and barriers to engagement with evidence-based practice among medical and dental trainees from the UK and Republic of Ireland: a national survey
Source: BMJ Open. 2019 Oct 17;9(10):e031809. doi: 10.1136/bmjopen-2019-031809 (PMC6803141; doi:10.1136/bmjopen-2019-031809)
Supplement: Supplementary data [file bmjopen-2019-031809supp002.pdf]

UK & Ireland Trainees Survey for Doctors & Dentists in Training

Cochrane UK & Ireland Trainees Advisory Group - Trainee Survey Working Group

Introduction

Thank you for completing this short survey, which should take no longer than 5 minutes for you to complete. It will ask you to report the extent to which you feel you practice evidence-based medicine or dentistry. The results will guide the Cochrane UK Trainees Advisory Group to develop resources that support you and other trainees in medicine and dentistry to apply the evidence base to your clinical work.

UK & Ireland Trainees Survey for Doctors & Dentists in Training
Cochrane UK & Ireland Trainees Advisory Group - Trainee Survey Working Group

Demographics

1. Which region are you currently training in?

2. Which specialty are you currently training in?
(choose N/A if you are a foundation trainee)

	Doctors	Dentists
Specialty		

Other (please specify)

3. Which stage of training pathway are you currently undertaking?

	Doctors	Dentists
Training stage		

Other (please specify)

4. Are you currently, or have you previously been in an academic training programme?

- Yes, currently
- Yes, previously
- No, never

5. If you have any additional qualification(s), please choose all appropriate. (If not, skip to the next page)

	Clinical	Research	Management/ Leadership	Statistics	Teaching	Other
Bachelors	☐	☐	☐	☐	☐	☐
Postgraduate Certificate	☐	☐	☐	☐	☐	☐
Postgraduate Diploma	☐	☐	☐	☐	☐	☐
Masters	☐	☐	☐	☐	☐	☐
Doctorate	☐	☐	☐	☐	☐	☐

Other (please specify)

UK & Ireland Trainees Survey for Doctors & Dentists in Training
Cochrane UK & Ireland Trainees Advisory Group - Trainee Survey Working Group

1. How frequently in your clinical practice would you refer to published literature* in order to determine the evidence-base for a specific action or intervention?

* Eg. Clinical Knowledge Summaries, NICE guidelines, systematic reviews, individual papers

- ○ Never
- ○ Less than once a month
- ○ More than once a month but not every week
- ○ At least once a week
- ○ On most days

2. Which of the following prevent you from more regularly using the evidence base in your clinical practice? (choose all applicable)

- ☐ Lack of relevant/ high quality evidence in my specialty
- ☐ Insufficient time to consult the literature
- ☐ Dfficult to access resources from my Trust
- ☐ Lack of confidence in directly basing my decisions on published findings
- ☐ I do not think evidence would change my clinical practice
- ☐ Difficulty in interpreting the statistics in the literature
- ☐ Tendency to follow the generally accepted clinical practice in my department
- ☐ Not relevant to me or my specialty (state which specialty in the box below)
- ☐ Other (please specify in the box below)
- ☐ None

Free text box

3. Which of the following motivate you to explore the evidence base to inform your clinical practice? (choose all applicable)

- ☐ Lack of a relevant guideline
- ☐ Poor quality guideline(s)
- ☐ Encouraged to by senior colleagues
- ☐ Routine practice in my specialty (state which specialty in the box below)
- ☐ Awareness of negative clinical outcome as a result of non-evidence based clinical practice
- ☐ Desire to better understand how clinical decisions are made
- ☐ I do not use the evidence base
- ☐ Other (please specify in the box below)

Free text box

4. Would you feel comfortable querying a colleague's management plan based on your reading of the evidence?

- ○ Yes - I would feel comfortable querying a colleague's management at all levels
- ○ Yes - but only if that colleague was more junior/ less experienced than me
- ○ No
- ○ Depends on the situation (please elaborate in the box below)

Comments

5. Which of the following sources of scientific evidence have you previously consulted to inform your clinical practice? (choose all applicable)

- ☐ None
- ☐ Published original research
- ☐ Narrative review articles
- ☐ Systematic reviews/ meta-analyses
- ☐ Conference abstracts
- ☐ NICE Clinical Knowledge Summary
- ☐ Local guidelines/ reports
- ☐ National guidelines
- ☐ Reports from a specialty-specific organisatoin

Other (please specify)

6. Have you previously consulted the Cochrane Library to seek evidence to solve a clinical problem?

- ○ Yes
- ○ No

7. How confident do you feel searching for evidence?

	1 (Not at all)	2	3	4	5 (Very confident)
Confidence level	○	○	○	○	○

8. How confident are you in interpreting basic statistics (e.g. confidence interval, relative risk) when reading a journal article?

	1 (Not at all)	2	3	4	5 (Very confident)
Confidence level	○	○	○	○	○

9. How would you use social media and the internet for your training/ education? (choose all applicable)

- [ ] Not be interested in using social media and the internet for this purpose
- [ ] I prefer offline materials (eg. offline courses, conference stands)
- [ ] I would be interested in reading an evidence-based practice Twitter feed that updated during the day
- [ ] I would use the Cochrane UK Facebook page
- [ ] I would use forums on the Cochrane UK website
- [ ] I would read a blog (eg. 'Evidently Cochrane')
- [ ] I would listen to a podcast
- [ ] I would be interested in receiving email updates on evidence-based practice from Cochrane UK for junior doctors/ dentists
- [ ] I would be interested in an online journal club to improve my critical appraisal skills and evidence-based practice
- [ ] Other (please specify)

10. Please leave any additional comments or your email address if you wish to be contacted to discuss survey-related topics. Thank you very much for your contribution to the survey.

Cochrane UK & Ireland Trainees Advisory Group - Trainees Survey Working Group

If you want to find out about opportuinties to get involved, please visit our website: uk.cochrane.org/trainees
