# Supplementary material for: Motivators and barriers to engagement with evidence-based practice among medical and dental trainees from the UK and Republic of Ireland: a national survey
Source: BMJ Open. 2019 Oct 17;9(10):e031809. doi: 10.1136/bmjopen-2019-031809 (PMC6803141; doi:10.1136/bmjopen-2019-031809)
Supplement: Supplementary data [file bmjopen-2019-031809supp001.pdf]

Motivators & Barriers to Engagement with Evidence Based Practice Amongst Medical & Dental Trainees from the United Kingdom & Republic of Ireland: a National Survey

Bosun Hong, Eoin O'Sullivan, Christin Henein, Christopher M. Jones

Supplementary Table 1. A summary of professional organisations contacted via email

Organisation / Specialty	Contact
**Deanery**	
All	hee.pressoffice@nhs.net
Health Education West Midlands	specialtyrecruitment@wm.hee.nhs.uk
Health Education East Midlands	communications.em@hee.nhs.uk
Health Education East of England	midlandsandeast.comms@nhs.net
Health Education North East	Enquiries.NE@hee.nhs.uk
Health Education North West	info.nw@hee.nhs.uk
Health Education Yorkshire & The Humber	contactus.yh@hee.nhs.uk
Health Education Thames Valley	enquiries.TV@hee.nhs.uk
Health Education Wessex	Reception.wx@hee.nhs.uk
Health Education South West	info.SW@hee.nhs.uk
Health Education South London	info.sl@hee.nhs.uk
Health Education North West London	info.nwl@hee.nhs.uk
Health Education North Central & East London	info.ncel@hee.nhs.uk
Health Education Kent, Surrey & Sussex	rotations.lase@hee.nhs.uk
Wales	walesdeanery@cardiff.ac.uk
**Royal College**	
Royal College of Anaesthetists	info@rcoa.ac.uk
Royal College of Emergency Medicine	No email
Royal College of General Practitioners	membership@rcgp.org.uk
Faculty of Intensive Care Medicine	ficm@rcoa.ac.uk
Royal College of Obstetricians & Gynaecologists	pressoffice@rcog.org.uk
Faculty of Occupational Medicine	Leigh.Harrison@FOM.ac.uk
Royal College of Paediatrics & Child Health	enquiries@rcpch.ac.uk
Royal College of Pathologists	info@rcpath.org
Faculty of Pharmaceutical Medicine	fpm@fpm.org.uk
Royal College of Physicians of Edinburgh	g.mcalister@rcpe.ac.uk
Royal College of Physicians of Ireland	No email
Royal College of Physicians of London	No email
Royal College of Physicians & Surgeons of Glasgow	No email
Royal College of Psychiatrists	reception@rcpsych.ac.uk
Faculty of Public Health	marijanacuric@fph.org.uk
Royal College of Radiologists	nquiries@rcr.ac.uk
Faculty of Sexual & Reproductive Health	No email
Royal College of Surgeons of Edinburgh	mail@rcsed.ac.uk
Royal College of Surgeons of England	communications@rcseng.ac.uk
Royal College of Surgeons of Ireland	communications@rcsi.ie
Faculty of Forensic and Legal Medicine	forensic.medicine@fflm.ac.uk

Hong B, *et al. BMJ Open* 2019; 9:e031809. doi: 10.1136/bmjopen-2019-031809

**Specialty**	
Acute Medicine	gg-uhb.CTMedicineScot@nhs.net<br>Ct1recruitment@jrcptb.org.uk<br>nationalrecruitment@nhslothian.scot.nhs.uk<br>st3recruitment@jrcptb.org.uk
Anaesthetics	edemployment.tayside@nhs.net<br>Medicalrecruitment.tayside@nhs.net<br>nationalrecruitment@wm.hee.nhs.uk
Cardiology	national.recruitment@nhslothian.scot.nhs.uk<br>St3recruitment@jrcptb.org.uk
Cardiothoracic surgery	nationalrecruitment@nes.scot.nhs.uk
Chemical Pathology ST1	nationalrecruitment@nes.scot.nhs.uk
Clinical Genetics	nationalrecruitment@nes.scot.nhs.uk<br>St3recruitment@jrcptb.org.uk
Clinical Pharmacology	nationalrecruitment@nes.scot.nhs.uk<br>St3recruitment@jrcptb.org.uk
Clinical Radiology	nationalrecruitment@nes.scot.nhs.uk
Core Anaesthesia	edeployment.tayside@nhs.net<br>nationalrecruitment@wm.hee.nhs.uk
Core Medicine	gg-uhb.CTMedicineScot@nhs.net<br>Ct1recruitment@jrcptb.org.uk
Core Psychiatry	nhsg.scotmtenquiries@nhs.net<br>Nwd.psychiatryrecruitment@nw.hee.nhs.uk
Core Surgical Training	nationalrecruitment@nes.scot.nhs.uk
Dermatology	nationalrecruitment@nes.scot.nhs.uk
Emergency Medicine	ACCSEMrecruitment@nes.scot.nhs.uk
Endocrine	st3recruitment@jrptb.org.uk
Gastroenterology	st3recruitment@jrcptb.org.uk
General Surgery	nationalrecruitment@nes.scot.nhs.uk
Genitourinary medicine	nationalrecruitment@nes.scot.nhs.uk
Geriatric medicine	st3recruitment@jrcptb.org.uk
General Practice	gpstp@nes.scot.nhs.uk
Haematology	st3recruitment@jrcptb.org.uk
Histopathology	nationalrecruitment@nes.scot.nhs.uk
Intensive care medicine	icm@wm.hee.nhs.uk
Medical oncology	nationalrecruitment@nes.scot.nhs.uk<br>st3recruitment@jrcptb.org.uk
Neurology	nationalrecruitment@nes.scot.nhs.uk<br>st3recruitment@jrcptb.org.uk
Neurosurgery	nationalrecruitment@nes.scot.nhs.uk
Obstetrics & Gynaecology	nationalrecruitment@nes.scot.nhs.uk<br>obsjobs@nw.hee.nhs.uk<br>obsandgynscotmt.recruitment@nhs.net
Ophthalmology	vana.fadda@rcophth.ac.uk
Otolaryngology	st3recruitment@jrptb.org.uk
Paediatrics	aa-uhb.clinicalsmtpaeds-crosshouse@nhs.net<br>paedjobs@rcpch.ac.uk
Paediatric cardiology	nationalrecruitment@nes.scot.nhs.uk
Public Health	nationalrecruitment@nes.scot.nhs.uk
Trauma & Orthopaedics	nhshighland.st1traumaorthopaedics@nhs.net
Sexual health	specialtyofficer@fsrh.org

Hong B, *et al. BMJ Open* 2019; 9:e031809. doi: 10.1136/bmjopen-2019-031809
