# Supplementary material for: Motivators and barriers to engagement with evidence-based practice among medical and dental trainees from the UK and Republic of Ireland: a national survey
Source: BMJ Open. 2019 Oct 17;9(10):e031809. doi: 10.1136/bmjopen-2019-031809 (PMC6803141; doi:10.1136/bmjopen-2019-031809)
Supplement: Author's manuscript [file bmjopen-2019-031809.draft_revisions.pdf]

BMJ Open

BMJ Open is committed to open peer review. As part of this commitment we make the peer review history of every article we publish publicly available.

When an article is published we post the peer reviewers' comments and the authors' responses online. We also post the versions of the paper that were used during peer review. These are the versions that the peer review comments apply to.

The versions of the paper that follow are the versions that were submitted during the peer review process. They are not the versions of record or the final published versions. They should not be cited or distributed as the published version of this manuscript.

BMJ Open is an open access journal and the full, final, typeset and author-corrected version of record of the manuscript is available on our site with no access controls, subscription charges or pay-per-view fees (http://bmjopen.bmj.com).

If you have any questions on BMJ Open's open peer review process please email info.bmjopen@bmj.com

BMJ Open

Motivators & Barriers to Engagement with Evidence Based Practice Amongst Medical & Dental Trainees from the United Kingdom & Republic of Ireland: a National Survey

Journal:	*BMJ Open*
Manuscript ID	bmjopen-2019-031809
Article Type:	Research
Date Submitted by the Author:	20-May-2019

Complete List of Authors:	Hong, Bosun; Birmingham Dental Hospital; University of Birmingham<br>O'Sullivan, Eoin; Renal Medicine<br>Henein, Christin; University of Newcastle upon Tyne; Moorfields Eye Hospital NHS Foundation Trust<br>Jones, Christopher; University of Leeds, ;

Keywords:	MEDICAL EDUCATION & TRAINING, Postgraduate Education, Evidence-based practice

SCHOLARONE™
Manuscripts

Motivators & Barriers to Engagement with Evidence Based Practice Amongst Medical & Dental Trainees from the United Kingdom & Republic of Ireland: a National Survey

Bosun Hong (bosunhong@gmail.com)[1,2,3], Eoin O'Sullivan (eoindosullivan@gmail.com)[1,4], Christin Henein (christin.henein@gmail.com)[1,5,6] & Christopher M. Jones (c.jones1@leeds.ac.uk)[1,7,8]

[1]Cochrane UK & Ireland Trainees Advisory Group; [2]Birmingham Dental Hospital, Birmingham Community Healthcare NHS Foundation Trust, UK; [3]School of Dentistry, College of Medical & Dental Sciences, University of Birmingham, UK; [4]MRC Centre for Inflammation Research, Queen's Medical Research Institute, University of Edinburgh, UK; [5]Institute of Genetic Medicine, Newcastle University, UK, [6]National Institute for Health Research Biomedical Research Centre at Moorfields Eye Hospital NHS Foundation Trust and UCL Institute of Ophthalmology, UK; [7]Faculty of Biological Sciences, University of Leeds, UK; [8]Radiotherapy Research Group, Leeds Cancer Centre, The Leeds Teaching Hospitals NHS Trust, UK.

Short title: UK & Ireland Medical & Dental Trainee Engagement with Evidence Based Practice

Keywords: Evidence-based practice, Evidence-based medicine, Postgraduate education

Word count: 4,003 words **Number of pages:** 19
Number of tables: 2 **Number of figures:** 4
Supplementary files: 2

Funding: This research received no specific grant support from the public, commercial or not-for-profit sectors. BH was supported for the duration of this work by a National Institute for Health Research (NIHR) Academic Clinical Fellowship (ACF) in Oral Surgery. EOS was supported for the duration of this work by Kidney Research UK. CH was supported for the duration of this work by a NIHR ACF in Ophthalmology. CMJ was supported for the duration of this work by a NIHR ACF in Clinical Oncology and a Wellcome Trust N4 Clinical Research Training Fellowship (203914/Z/16/Z).

Competing interests: The authors declare that they have no relevant conflicts of interest.

Data sharing statement: Data available on request

Address all correspondence and requests for reprints to:
Christopher M. Jones
Level 7 LIGHT Laboratories
Faculty of Biological Sciences
University of Leeds
Leeds
LS2 9JT, UK.

For peer review only - http://bmjopen.bmj.com/site/about/guidelines.xhtml

ABSTRACT

Objectives: To explore the extent to which doctors and dentists in training within the United Kingdom (UK) and Republic of Ireland (RoI) engage in and with evidence based practice (EBP), and to identify motivators and barriers to them doing so.

Design: An observational, pre-piloted web-based survey developed by a trainee-led focus group.

Setting: The survey instrument was disseminated to doctors and dentists in training within the UK and RoI during June 2017 via social media and through Deanery, Royal College and specialty-specific mailing lists.

Participants: Data from 243 trainees were analysed; 188 doctors from 31 specialties and 55 dentists from nine specialties. Responses were received from trainees at all stages of postgraduate training.

Primary & secondary outcome measures: The extent to which doctors and dentists in training engage with EBP and the motivators and barriers to them doing so; as assessed via a mixed methods approach using both quantitative and qualitative analyses.

Results: Cronbach's α was 0.83. Most trainees (87.6% of doctors and 75.1% of dentists) consulted the evidence base at least monthly, whilst 23.1% of both specialties did so daily. The two most commonly cited barriers to engagement with EBP for both doctors and dentists respectively were insufficient time (57.6% and 45.1%) and a tendency to follow departmental practice (40.6% and 45.1%). Key motivators for EBP included curiosity, following the example set by senior colleagues and a desire to avoid harm. Most trainees reported high levels of confidence interpreting evidence yet for 19.6% of doctors and 30.8% of dentists, medical hierarchy would impede them querying a colleague's management plan based on their own reading of the evidence.

Conclusions: Time, accepted departmental practice and the behaviour of senior clinicians all highly impact on trainee engagement with EBP. Policy makers and healthcare organisations should engender cultures in which EBP is encouraged.

For peer review only

ARTICLE SUMMARY

Strengths & limitations of this study

1. National survey detailing medical and dental trainees' level of engagement with evidence-based practice.

2. Provides policy makers, trainers and healthcare organisations with evidence for areas in which trainees' engagement with evidence-based practice can be encouraged and facilitated.

3. Reach extends across the UK and Republic of Ireland but results may not represent trainees' experiences in other countries.

4. Both dentists and doctors in training sampled but modest sample size may limit generalisability.

For peer review only

For peer review only - http://bmjopen.bmj.com/site/about/guidelines.xhtml

INTRODUCTION

Evidence based practice (EBP) describes the integration of a patient's values and a clinician's expertise with the contemporary scientific evidence base.(1) In doing so it prioritises the implementation of knowledge gained through systematic scientific discovery above that of anecdotal physician experience, reducing the potential impact of physician bias whilst empowering all clinicians, including those with less experience such as trainees, to make standardised, cost effective decisions informed by high-quality evidence.(2) There is significant evidence for the overall benefit of implementing EBP.(3-5)

Within the United Kingdom (UK) and the Republic of Ireland (RoI), EBP is firmly embedded within both medical and dental curricula. Reflecting this, the General Medical Council recommends that new doctors must be able to 'access and analyse reliable sources of current clinical evidence and guidance and have established methods for making sure their practice is consistent with these'.(6) Similarly, the General Dental Council stipulates that upon registration, dental trainees should be able to apply an evidence-based approach and utilise critical thinking skills.(7)

Despite these recommendations and the known tangible benefits of EBP to patient outcomes and care, it is recognised that clinical practice is not universally 'evidence based'.(3, 4, 8) The barriers to EBP have been studied previously and are numerous.(9-11) Commonly cited examples include inadequate knowledge or awareness of relevant evidence, a lack of individual motivation to engage with the evidence base and difficulty reconciling available evidence with a clinical question.(9)

The extent to which these barriers specifically apply to postgraduate medical and dental trainees, and the degree to which they therefore engage with EBP, is for the most part unknown. Much of the evidence cited to date is instead drawn from grouped populations of undergraduate and postgraduate trainees, or from cohorts consisting of both trainees and more senior clinicians who are beyond completion of training.(10, 12) The characteristics of the working environment, clinical responsibilities and drivers for learning of postgraduate trainees may differ from those of non-training clinicians or undergraduates. Hence, an understanding of the role the postgraduate training environment plays in moulding trainees'

For peer review only - http://bmjopen.bmj.com/site/about/guidelines.xhtml

desire and ability to engage with the evidence base is important. It may, for instance, guide regulators and educators alike in designing interventions to promote the translation of EBP teaching into clinical work for this population. It may also provide training programme coordinators and senior clinicians with an insight into how best to support their more junior colleagues to engage in EBP.

The Cochrane UK & Ireland Trainees Advisory Group was established as the trainee-led arm of Cochrane UK & Ireland in 2016. It is formed of 15 medical and dental trainees from across the UK & RoI who represent a diverse range of specialties and who are together tasked with enhancing trainee-engagement with EBP. Given the lack of evidence concerning trainees' engagement with EBP in the contemporary UK and RoI healthcare systems, the Trainees Advisory Group undertook a national survey focused on trainees' use of the evidence base in routine clinical practice and the wider motivators for and barriers to use of the evidence base amongst these doctors and dentists in training.

METHODS

Study design

We conducted a cross-sectional study targeting doctors and dentists in postgraduate training from across the UK and RoI using an anonymous electronic survey between June and October 2017.

Survey instrument

Members of the Cochrane UK & Ireland Trainees Advisory Group formed a focus group in order to develop an online survey specifically for this study. Drawing on their own experiences, the Trainees Advisory Group defined the following as areas of focus for the survey: (a) trainee engagement with EBP, (b) motivators and barriers to engagement with EBP; and (c) trainees' preferences towards approaches to enhance engagement in EBP. Engagement in this context was defined as maintaining sufficient knowledge of the contemporary evidence base relevant to one's clinical role to influence views of or specific practice within that role.

For peer review only - http://bmjopen.bmj.com/site/about/guidelines.xhtml

The survey instrument was devised and refined through an iterative process that included two pilot surveys with 19 postgraduate trainees who represented 13 specialties in the fields of medicine and dentistry, and seven geographical locations within the UK and RoI. In its final form (**Supp. Materials**) the survey instrument consisted of 14 closed-ended questions, five of which also permitted optional free-text response and two of which incorporated a five-point Likert scale. Included questions focussed on the aforementioned areas of interest defined by the focus group. Demographic data were also collected and included each trainee's specialty and training stage, prior academic training and their location, subdivided as either RoI or by UK deanery. We collected data using a cloud-based online survey programme (SurveyMonkey, California, USA).

Study population & survey dissemination

Trainees in a recognised UK or RoI training post within any medical or dental specialty formed the target population for this study. Full registration with the General Medical Council was not required in order to ensure that responses from Foundation Year 1 doctors were considered. UK and RoI trainees are widely geographically distributed and are overseen by a large number of professional organisations, including regional deaneries and various Royal Colleges. There is no single point of contact for either doctors or dentists in training. In order to enhance coverage of trainees eligible to participate in this survey, we used a phased approach to its dissemination that over a three-week period incorporated both a social media arm and direct approaches to trainees via email and online training portfolios (see **Fig. 1**).

In week one (commencing 30/06/2017), we disseminated the survey via email to relevant professional organisations with oversight of doctors and dentists in training across the UK and RoI, and by using the email marketing service MailChimp® to medical deaneries across the UK and to 49 trainees subscribed to the Cochrane UK & Ireland Trainees Advisory Group newsletter (see **Supp. Table 1**). We also distributed to UK dental deaneries following approval from the UK Committee of Postgraduate Dental Deans and Directors (COPDEND). In both instances, administrators within these professional bodies

For peer review only - http://bmjopen.bmj.com/site/about/guidelines.xhtml

were asked to distribute the survey to trainees overseen by their organisation via direct email or through use of the NHS training ePortfolio, to which all medical trainees have access.

During this same week, a link to the survey was posted online using the Cochrane UK Twitter account (@CochraneUK), which at the time had in excess of 40,000 followers. In line with an *a priori* planned social media strategy, this link was re-tweeted nine times by this same account and by members of the Cochrane UK & Ireland Trainees Advisory Group within the same week. Tweets were flagged for the attention of the Twitter accounts of relevant Royal Colleges and other bodies overseeing trainees.

In week two, we once again contacted those professional bodies who had been contacted during week one but who had not responded to the first survey approach. Contemporaneously, further Tweets outlining the survey were distributed by the Cochrane UK & Ireland Twitter account. These continued into the third week. The survey subsequently remained open until October 2017 in order to permit further responses to be collected.

Data analysis

The unit of analysis of this study is the individual respondent (trainee). Data are provided for doctors and dentists separately where possible. There were insufficient participant numbers to analyse by specialty. Data are shown for separate stages of training where numbers allow. Descriptive analyses were undertaken using a statistical software, R (R Core Team, version 3.4.3, 2013). We computed the frequency of answers and where relevant expressed this as a percentage of total respondents. For likert-type scaled response answers, we assumed answers to be an interval-level measurement to allow visualisation using bar plots.

We conducted a thematic analysis of the free text responses using an inductive approach, following the phases of thematic analysis outlined by Braun and Clarke.(13) Having become familiarised with the data, we identified initial codes using a qualitative data analysis software (NVivo, QSR International Pty Ltd., version 12, 2018) and organised the codes into potential themes. The identified themes were

For peer review only - http://bmjopen.bmj.com/site/about/guidelines.xhtml

reviewed and refined before final categorisation. In addition, we have searched for and retained deviant responses that do not fit into the themes but nevertheless make a valuable contribution to the overall message of this work.

Ethical approval

There was no requirement for specific ethical approval for this study. Consent to participate was implicit from the completion of the study survey and all potential participants were reminded that they were under no obligation to complete the survey. There was no incentive offered for survey completion.

Patient & public involvement

There was no direct patient involvement in the design or conduct of this study.

RESULTS

Internal consistency

The survey had high internal consistency with a Cronbach's α of 0.83.

Respondent demographics

A total of 243 responses were received; 188 (83.3%) from doctors in 31 specialties, and 55 (16.7%) from dentists in 9 specialties (see **Table 1**). Most (53%) trainees were specialty training 3 (ST3) level or above in seniority, and the majority (70%) had not previously undertaken formal postgraduate academic training. Responses were received from trainees in RoI and in 20 out of 21 UK deaneries. The geographical distribution of respondents is summarised in **Table 2.**

Engagement with EBP

To assess trainees' level of engagement with EBP, those responding to the survey were asked how frequently they would refer to published literature in order to determine the evidence base for a specific action or intervention (**Fig. 2a**). The majority of doctors (87.6%) and dentists (75.1%) reported

For peer review only

For peer review only - http://bmjopen.bmj.com/site/about/guidelines.xhtml

consulting the evidence base at least monthly. An identical proportion (23.1%) of the two professions consulted the evidence base daily.

Trainees were additionally asked to report which sources of evidence they had previously consulted in order to inform their practice (**Fig. 2b**). For both doctors and dentists, national (95.8% and 88.5% respectively) and local (81.4% and 67.3%) guidance, NICE Clinical Knowledge Summaries (82.6% and 86.5%), systematic reviews (85.6% and 84.6%) and published original research (79.6% and 63.5%) formed the principal sources of information.

Trainees' self-ratings of their confidence in searching evidence and interpreting basic statistics were strongly negatively skewed (**Figs. 3 a** and **b)**, though this distribution became normal when trainees with prior academic experience were removed from the analysis. As highlighted by **Figs. 3**, confidence in these key skills also increases with training stage.

When asked for their perception of social media and the internet for education to support their EBP, the majority of respondents (73.2%) were supportive of its use. Popular forms of engagement were via email updates (53.7%), an online journal club (47.3%) and through a podcast (40.4%).

Motivators for EBP

There was some variation between doctors and dentists in the factor respondents highlighted as their motivation for consulting the evidence base (**Fig. 4a**). For dentists, the most frequently cited reason was to better understand how clinical decisions are made (84.3%), a curiosity noted by 54.6% of responding doctors. Conversely, whilst 42.4% of doctors reported that consulting the evidence base was routine practice in their specialty, this was the case for just 17.6% of dentists. For both doctors and dentists, lack of a relevant guideline (37.0% and 39.2% respectively), encouragement from senior colleagues (37.0% and 45.1%) and an awareness of the potential for a negative clinical outcome from not consulting the evidence base (34.6% and 41.2%) were other frequently reported motivators for engagement in EBP.

For peer review only - http://bmjopen.bmj.com/site/about/guidelines.xhtml

For peer review only

These findings were supported by thematic analysis of free-text comments, which revealed that motivators for EBP included trainees' desire to provide a good standard of care at either the individual patient level, or for public health trainees, at the population level. The responses captured the inspiration to do so, which included examples of harm caused to patients by non-EBP, involvement in research projects and following the example set by senior colleagues:

> "I think it is important to review literature as often ingrained practice has no real proof of benefit. I also think that by understanding the evidence you learn better and you understand its applicability and are better able to decide if it applicable for your patient cohort."

> "The ethical implications of continuing to practice in an evidence-light environment following the withdrawal of the Liverpool Care Pathway"

> "…My consultants are also very much into [EBP] & quote papers all the time. I feel that I'm expected to know the evidence behind the treatments I offer patients & I think this is a good thing as it makes me much more confident at recommending a particular treatment to a patient."

Barriers to engagement with EBP

Time, access & local practices

Barriers to EBP reported by trainees are summarised in **Fig. 4b**. The two most commonly cited barriers for doctors and dentists respectively were insufficient time (57.6% and 45.1%) and a tendency to follow departmental practice (40.6% and 45.1%). Limited access to relevant evidence from the workplace was also commonly reported as a barrier, though more frequently amongst doctors (40.6%) than dentists (25.5%).

Thematic analysis of free-text responses supported these data, and highlighted that trainees considered time and access as important barriers to EBP:

For peer review only - http://bmjopen.bmj.com/site/about/guidelines.xhtml

> "By far the greatest barrier to taking an evidence-based approach to my work is the lack of access to the evidence. I am a public health registrar with a passion for the wider determinants of health. At the moment I have very limited Athens access through the training scheme, but following this I'm not sure if I will even have that…"

> "I am limited by the insufficient electronic journal access provided as part of NHS Athens, which is inferior to the access provided by a good university account. I do manage to work around this, but it takes extra time and limits the sources I can use"

Some trainees felt that the access issue was two-fold; electronic access to articles being the first hurdle and having the information technology facility within the NHS premises to allow access to the information being the next one:

> "…the evidence base is spread across multiple resources, some of which are, with the best will in the world difficult to use in day to day practice, from crappy, locked down NHS computers. If they are free to use at all."

> "Poor wifi signal often frustrates me when I want to look at a resource such as NICE or UptoDate on the wards"

Some trainees felt that the use of evidence was not prioritised in postgraduate training despite its importance, and that those who require further training on this may struggle to find the time due to other priorities:

> "We are not taught how to use the evidence base. Yeah sure, the local library might offer some introductory courses (if you are in a good hospital) but it is unacceptable that 'moving &

For peer review only

handling' is a mandatory course but searching and using the evidence based is treated as optional."

"I was lucky enough to learn about evidence-based research at medical school and taught how to conduct literature reviews but it is something I just don't have the time for now."

Limited evidence

Dentists in training more commonly cited a lack of relevant or high-quality evidence as a barrier than doctors (37.3% vs 12.7%). Thematic analysis also revealed the challenges trainees face in specialties in which evidence is scarce and where studies have unclearly reported methodology or questionable generalisability to their patients:

"…there is a really lack of randomised controlled trials in my specialty and rejection of them by some of the large research groups. The question of how much evidence is enough evidence in palliative medicine remains to be answered."

Reliance on secondary evidence such as local, specialty-specific, or national guidelines was evident in the free-text responses. Trainees perceived secondary evidence to be of high quality and therefore their default first choice of avenue for evidence:

"I use individual papers very rarely. Think Royal College of Obstetrics and Gynaecology does a good job of synthesising current information and producing guidance on common topics."

"commonly used guidelines have been memorised – e.g. asthma, transient loss of consciousness"

For peer review only

Hierarchy

When trainees were asked if they would feel comfortable querying a colleague's management plan based on the trainee's reading of the evidence, 57.7% of doctors and 51.9% of dentists were unconditionally so. Other trainees reported that they would feel confident only if the colleague was more junior or less experienced than them (19.6% and 30.8%), or that this depended on the situation (15.5% and 11.5%). A smaller proportion of respondents stated that they would feel uncomfortable about querying a colleague's management in any case (7.1% and 5.8%).

Upon analysing the free-text responses, a passive attitude of following generally accepted departmental practice was a frequent feature. It was evident that the working relationship with, or the personality of a senior colleague was more important to trainees than the seniority itself when considering whether to question a senior colleague's decision. Embedded within the responses was a situational analysis process that trainees go through in their mind before deciding to approach their senior colleague. Some trainees have developed a strategy where their decision to question a senior colleague would be conditional – for example, only if the impact to the patient is large, only if they predict a positive response from the colleague, only if they have direct responsibility for care (i.e. "asked to complete the treatment"):

> "Some colleague's personalities may colour my comfort level to query/discuss management!"

> "Consultants who are confident and progressive would be willing to discuss. Some seniors would find this threatening as they are insecure in their own practice."

> "…you have to read the situation…"

> "It really depends on so many factors (e.g. who is the manager, who are you, how long you have been there, what type of relation you have...) In general, I would only do it if I feel that

For peer review only

there will be a positive response. If I feel that it is only going to generate conflict, then, what's the point?"

"… I pick my fights as such and only raise it if I think it is important/going to make a difference etc."

EBM malaise

There was also a response which pointed out that a clinician's unfounded preconceived notions may act as a barrier to a scientific enquiry:

"The problem is that the concept of "evidence" has lost its meaning. People use for all sorts of things...I mean that people, when faced by a public health issue, they don't ask themselves "What does the evidence say about how to resolve this issue?". What usually happens is that they have a pre-defined position and they ask themselves "What is the evidence that supports my position?" That influences how searches are done, how bias is assessed and so on."

DISCUSSION

The extent to which doctors and dentists in contemporary training schemes engage with the evidence base, and the barriers and motivators to them doing so, is underexplored. We outline here a nationwide survey of UK and RoI doctors and dentists from a large number of specialties and at all stages of training. Most actively engage with literature at least monthly, whilst a quarter do so daily. Dentists in training most frequently cited curiosity as their main motivator for EBP and reported a lack of relevant evidence as a key barrier to further engagement with the evidence base. On the other hand, doctors frequently cited their engagement with EBP in the context of accepted practice for the departments in which they work. For both doctors and dentists, a lack of time and poor access to relevant literature were key barriers to further engagement in EBP. Trainees nevertheless reported high levels of confidence in searching for evidence and interpreting basic statistics. However, the majority also

For peer review only - http://bmjopen.bmj.com/site/about/guidelines.xhtml

reported that they would not query a more senior colleague based on their own reading of the evidence, thereby potentially limiting the extent to which their confidence translates in to EBP.

The notion that trainees appreciate EBP but do not consider their practice to be consistently evidence-based is supported elsewhere in the literature.(14-16) For instance, a survey of pre-registration house officers (currently termed foundation trainees in the UK) in the Mersey Deanery revealed that trainees considered critical appraisal skills to be relevant to their practice yet only just over half felt that their clinical practice was based on the best available evidence.(14) Similarly, a survey of obstetrics and gynaecology trainees in West Midlands revealed that whilst most trainees perceived EBP as a positive concept, relatively few consistently referred to literature.(15) It is of interest that many of the barriers identified in these more limited specialty-specific study are reflected in the broad trainee population reported here.

Our findings have a number of additional implications, which the Cochrane UK & Ireland Trainees Advisory Group have reviewed with a view to formulating strategic, concrete plan to promote EBP. The higher reported confidence in EBP of trainees who have undertaken formal postgraduate academic training demonstrates the value of academic training schemes to healthcare settings. Notably, that the hierarchy and departmental practice so considerably shape the degree to which trainees engage in EBP demonstrates the parallel importance of ensuring an appropriate training environment. In view of this, the Trainees Advisory Group plans to collect examples of good practice as a result of postgraduate academic training and positive influence from colleagues or department practice, and disseminate them through its online platforms. The data depicted in our survey additionally demonstrate a clear need to support busy trainees in digesting evidence, whether through directly managing their workload or by ensuring that sources of evidence are readily available and their conclusions efficiently consumable. Other implications of this work are likely to be harder to address. The paucity of relevant evidence highlighted by dental trainees is one such area but may be challenged by promoting greater awareness of EBP within the profession.

For peer review only - http://bmjopen.bmj.com/site/about/guidelines.xhtml

Limitations

The number of trainees reached by the survey is not known but the overall response rate is low in the context of the thousands of doctors and dentists in training within the UK and RoI. The degree to which the data depicted here represent the overall population is, therefore, unclear and the data are prone to responder bias. Further, a number of specialties were not represented or were but by only a small number of respondents, and other specialties such as public health were relatively over-represented. As such, it is impossible to characterise differences in the data between specialties and training stages. The use of email and social media to contact doctors and dentists also provides the potential for selection bias. The extent to which these results are applicable to healthcare settings outside of the UK and RoI is in addition unclear.

This survey nevertheless had a wide geographical reach across both the UK and RoI, incorporated a significant number of specialties and features responses from a large number of doctors and dentists in training. Internal consistency was also high, as measured by a Cronbach's α of 0.83. The data provide a number of clear themes, some of which are specific to the postgraduate trainee population. For example, the important influence of hierarchy, both as a motivator and a barrier, is a trainee-specific theme that emerged prominently as a result of our focus on the postgraduate trainee population. These themes provide a starting point for further research and intervention from both healthcare professionals and policymakers. The validity of these themes is supported by their concordance with historical evidence (14-16), and their relevance is reflected from the post-modernising medical careers cohort from which they are derived.

CONCLUSION

Time and a lack of access to key resources limit the ability of doctors and dentists in training within the UK and RoI to engage with EBP. Whilst the majority of these trainees report high levels of confidence in searching and interpreting statistics in the literature, a significant proportion highlight that hierarchy and departmental practice significantly shape their EBP. The extent to which these data vary between specialties and by stage of training is unknown and requires further research. Healthcare professionals

For peer review only - http://bmjopen.bmj.com/site/about/guidelines.xhtml

and policymakers should ensure that trainees are provided with the time and ability to efficiently access sources of evidence, and should engender cultures in which EBP is encouraged.

AUTHOR CONTRIBUTIONS

BH and CMJ devised, piloted and carried out the survey, and collated data. BH prepared the first draft of the manuscript. BH, EOS and CMJ led the analysis of data. All authors contributed to the interpretation of results and revisions of the draft manuscript. All authors have read and approved the final version of the manuscript.

ACKNOWLEDGEMENTS

We are grateful to trainees for taking the time to respond to this survey and to the various professional bodies who distributed the survey. We are also grateful for support from Cochrane UK, which is funded by the National Institute for Health Research (NIHR), and to Dr Emma Plugge for her critical appraisal of the manuscript.

For peer review only

REFERENCES

1. Sackett DL, Rosenberg WM, Gray JA, Haynes RB, Richardson WS. Evidence based medicine: what it is and what it isn't. 1996. Clin Orthop Relat Res. 2007;455:3-5.
2. Lewis SJ, Orland BI. The importance and impact of evidence-based medicine. J Manag Care Pharm. 2004;10(5 Suppl A):S3-5.
3. Lenfant C. Shattuck lecture--clinical research to clinical practice--lost in translation? N Engl J Med. 2003;349(9):868-74.
4. Awtry EH, Loscalzo J. Aspirin. Circulation. 2000;101(10):1206-18.
5. Stafford RS, Radley DC. The underutilization of cardiac medications of proven benefit, 1990 to 2002. J Am Coll Cardiol. 2003;41(1):56-61.
6. The General Medical Council. Outcomes for graduates. London, UK; 2018
7. The General Dental Council. Preparing for practice: dental team learning outcomes for registration. 2012.
8. O'Connor GT, Quinton HB, Traven ND, Ramunno LD, Dodds TA, Marciniak TA, et al. Geographic variation in the treatment of acute myocardial infarction: the Cooperative Cardiovascular Project. JAMA. 1999;281(7):627-33.
9. Cabana MD, Rand CS, Powe NR, Wu AW, Wilson MH, Abboud PA, et al. Why don't physicians follow clinical practice guidelines? A framework for improvement. JAMA. 1999;282(15):1458-65.
10. Taylor R, Reeves B, Ewings P, Binns S, Keast J, Mears R. A systematic review of the effectiveness of critical appraisal skills training for clinicians. Med Educ. 2000;34(2):120-5.
11. Hannes K, Norre D, Goedhuys J, Naert I, Aertgeerts B. Obstacles to implementing evidence-based dentistry: a focus group-based study. J Dent Educ. 2008;72(6):736-44.
12. Sullivan KJ, Wayne C, Patey AM, Nasr A. Barriers and facilitators to the implementation of evidence-based practice by pediatric surgeons. J Pediatr Surg. 2017;52(10):1666-73.
13. Clarke V, Braun V, Delgadillo J. Using thematic analysis in counselling and psychotherapy research: A critical reflection2018.
14. Doran T, Maudsley G, Zakhour H. Time to think? Questionnaire survey of pre-registration house officers' experiences of critical appraisal in the Mersey Deanery. Med Educ. 2007;41(5):487-94.
15. Awonuga AO DL, Khan KS, Taylor R. Postgraduate obstetrics and gynaecology trainees' views and understanding of evidence-based medicine. Med Teach. 2000;22:27-9.
16. Manikam L HA, Fosker H, Wong MHY, Banerjee J, Lakhanpaul M, Knight A, Littlejohns P. What drives junior doctors to use clinical practice guidelies? A national cross-sectional survey of foundation doctors in Engalnd & Wales. BMC Med Educ. 2015;15:227.

For peer review only - http://bmjopen.bmj.com/site/about/guidelines.xhtml

FIGURE LEGENDS

Figure 1: A flowchart depicting the survey dissemination approach, including via social media.

Figure 2: Trainee engagement with evidence based practice, including **(a)** the frequency with which trainees consult published literature in order to evaluate a clinical problem and **(b)** the sources trainees would typically consult in order to assess the evidence base.

Figure 3: Perceived confidence in searching evidence **(a)** and interpreting basic statistics **(b)** on a scale of 1 (least confident) to 5 (most confident).

Figure 4: A summary of reported **(a)** motivators and **(b)** barriers to engagement with EBP.

For peer review only

For peer review only - http://bmjopen.bmj.com/site/about/guidelines.xhtml

TABLE 1: Survey respondents by profession and specialty.

	**Doctors** (n=188)		**Dentists** (n=55)	
	N	%	N	%
**Specialty**				
Acute internal medicine	1	0.5		
Anaesthetics	7	3.7		
Cardiology	1	0.5		
Chemical pathology	1	0.5		
Clinical radiology	2	1.0		
Community sexual and reproductive health	12	6.4		
Dermatology	2	1.1		
Emergency medicine	5	2.7		
Foundation training	24	12.8	27	49.1
General medicine	5	2.7		
General practice	6	3.2	1	1.8
General psychiatry	2	1.1		
General surgery	6	3.2		
Geriatric medicine	4	2.1		
Haematology	1	0.5		
Oral histopathology			1	1.8
Intensive care medicine	1	0.5		
Infectious diseases	2	1.1		
Neurosurgery	1	0.5		
Obstetrics & gynaecology	20	10.6		
Occupational medicine	2	1.1		
Ophthalmology	2	1.1		
Oral and maxillofacial surgery*	1	0.5	3	5.5
Oral medicine			8	14.5
Oral surgery			5	9.1
Orthodontics			2	3.6
Paediatric and perinatal pathology	1	0.5		
Paediatric dentistry			4	7.3
Paediatric medicine	9	4.8		
Palliative medicine	2	1.1		
Periodontology as a monospecialty			1	1.8
Plastic surgery	1	0.5		
Public health medicine	57	30.3		
Renal medicine	3	1.6		
Respiratory medicine	2	1.1		
Restorative dentistry			2	3.6
Special care dentistry			1	1.8
Urology	1	0.5		
Vascular surgery	1	0.5		
Unknown	3	1.6		

*Oral and maxillofacial surgery is recognised as a specialty by the General Medical Council and as such their specialty trainees are registrants of the General Medical Council. In addition to specialty training, the specialty facilitates dental core training and therefore the majority of their junior staff are dentists

For peer review only

TABLE 2: Survey respondents by profession, location of training and training stage.

	**Doctors** (n=188)		**Dentists** (n=55)	
	N	%	N	%
**Respondent's location of training**				
Scotland	13	6.9	25	45.5
East Midlands, England	9	4.8	6	10.9
East of England	5	2.7	3	5.5
London, England	33	17.6	2	3.6
North East, England	3	1.6	1	1.8
North West, England	10	5.3	4	7.3
South East, England	22	11.7	2	3.6
South West, England	17	9.0	2	3.6
West Midlands, England	15	8.0	6	10.9
Yorkshire and the Humber, England	19	10.1	1	1.8
Wales	4	2.1	0	0.0
Northern Ireland	3	1.6	0	0.0
Republic of Ireland	28	14.9	2	3.6
Unknown	7	3.7	1	1.8
**Training stage**				
Foundation years	22	11.7	28	50.9
Core/Specialty trainee 1/2	39	20.7	15	27.3
Specialty trainee 3	30	16.0	4	7.3
Specialty trainee 4	31	16.4	2	3.6
Specialty trainee 5	20	10.6	2	3.6
Specialty trainee 6	9	4.8	1	1.8
Specialty trainee 7	8	4.3	0	0.0
Specialty trainee 8	0	0.0	2	3.6
RoI – Intern	3	1.6	0	0.0
RoI – Senior House Officer	4	2.1	1	1.8
RoI – Specialty Registrar	10	5.3	0	0.0
Unknown	12	6.4	0	0.0

For peer review only

SUPPLEMENTARY MATERIAL

Supplementary Table 1: A summary of professional organisations contacted via email

Supplementary File: Web-based survey instrument.

For peer review only

For peer review only - http://bmjopen.bmj.com/site/about/guidelines.xhtml

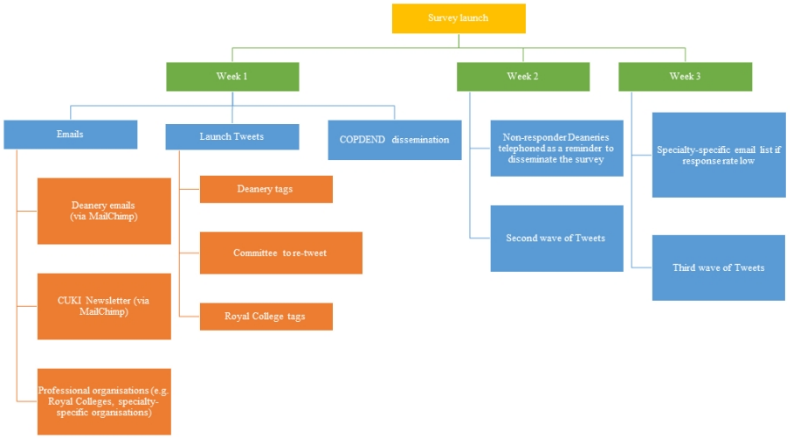

Figure 1: A flowchart depicting the survey dissemination approach, including via social media.

338x190mm (96 x 96 DPI)

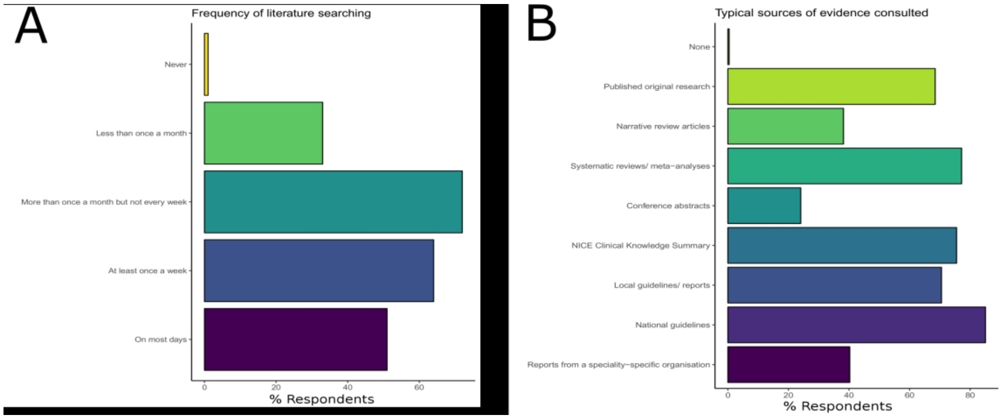

Figure 2: Trainee engagement with evidence based practice, including (a) the frequency with which trainees consult published literature in order to evaluate a clinical problem and (b) the sources trainees would typically consult in order to assess the evidence base.

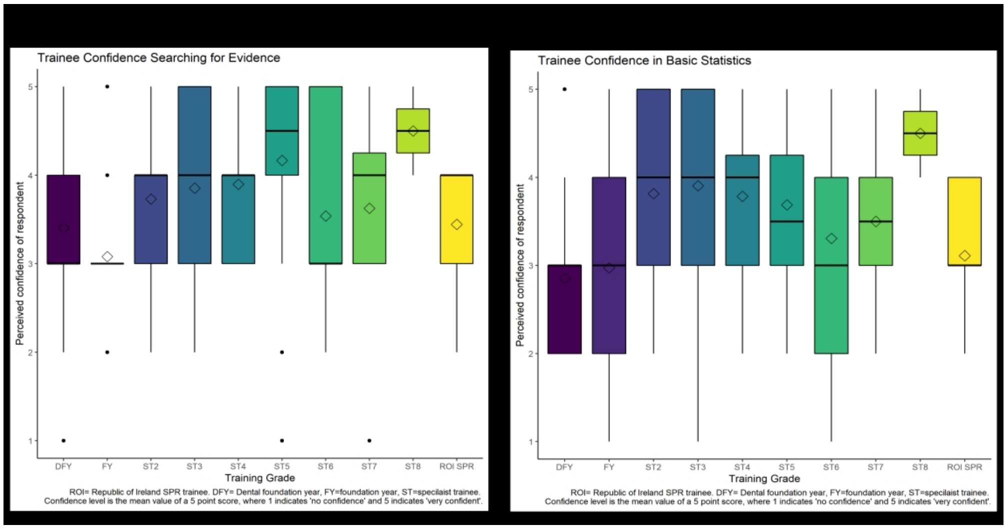

Figure 3: Perceived confidence in searching evidence (a) and interpreting basic statistics (b) on a scale of 1 (least confident) to 5 (most confident).

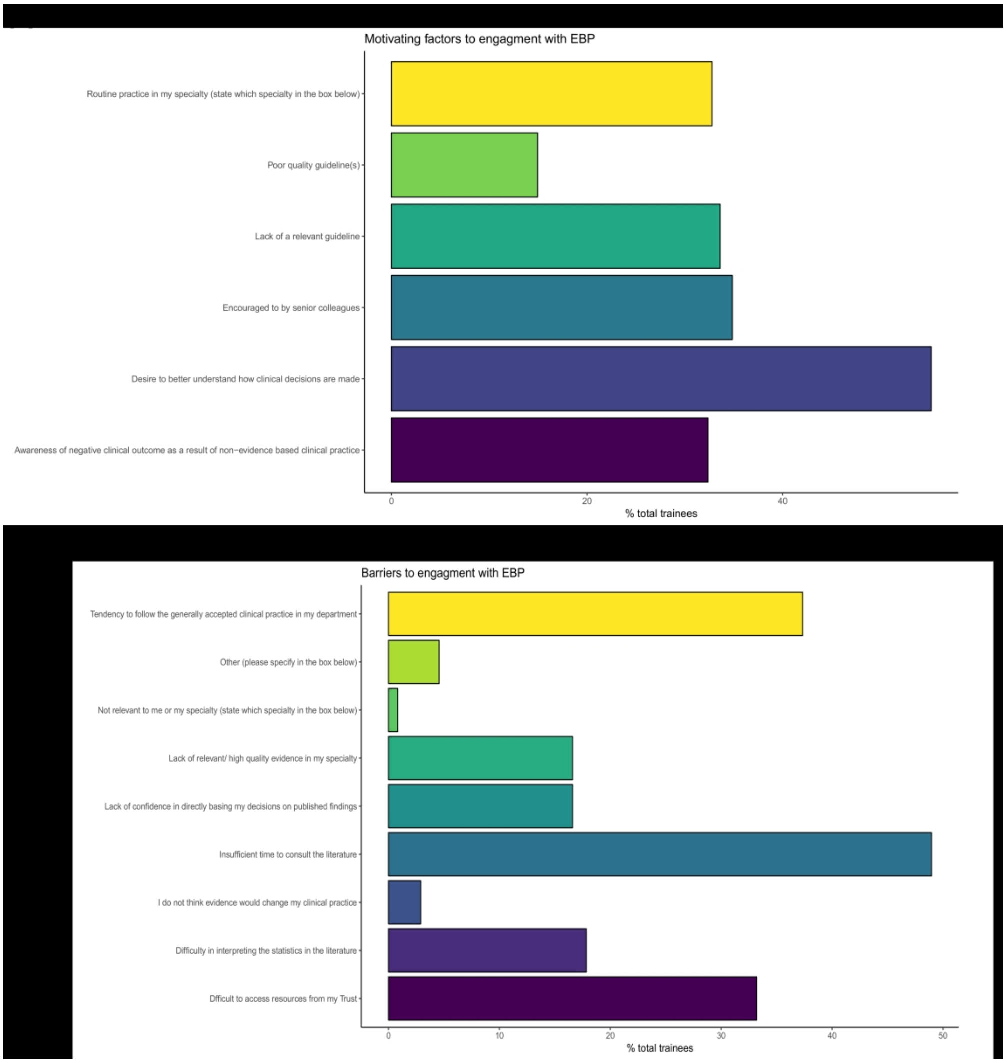

Figure 4: A summary of reported (a) motivators and (b) barriers to engagement with EBP.

Motivators & Barriers to Engagement with Evidence Based Practice Amongst Medical & Dental Trainees from the United Kingdom & Republic of Ireland: a National Survey

Bosun Hong, Eoin O'Sullivan, Christin Henein, Christopher M. Jones

Supplementary Table 1. A summary of professional organisations contacted via email

Organisation / Specialty	Contact
**Deanery**	
All	hee.pressoffice@nhs.net
Health Education West Midlands	specialtyrecruitment@wm.hee.nhs.uk
Health Education East Midlands	communications.em@hee.nhs.uk
Health Education East of England	midlandsandeast.comms@nhs.net
Health Education North East	Enquiries.NE@hee.nhs.uk
Health Education North West	info.nw@hee.nhs.uk
Health Education Yorkshire & The Humber	contactus.yh@hee.nhs.uk
Health Education Thames Valley	enquiries.TV@hee.nhs.uk
Health Education Wessex	Reception.wx@hee.nhs.uk
Health Education South West	info.SW@hee.nhs.uk
Health Education South London	info.sl@hee.nhs.uk
Health Education North West London	info.nwl@hee.nhs.uk
Health Education North Central & East London	info.ncel@hee.nhs.uk
Health Education Kent, Surrey & Sussex	rotations.lase@hee.nhs.uk
Wales	walesdeanery@cardiff.ac.uk
**Royal College**	
Royal College of Anaesthetists	info@rcoa.ac.uk
Royal College of Emergency Medicine	No email
Royal College of General Practitioners	membership@rcgp.org.uk
Faculty of Intensive Care Medicine	ficm@rcoa.ac.uk
Royal College of Obstetricians & Gynaecologists	pressoffice@rcog.org.uk
Faculty of Occupational Medicine	Leigh.Harrison@FOM.ac.uk
Royal College of Paediatrics & Child Health	enquiries@rcpch.ac.uk
Royal College of Pathologists	info@rcpath.org
Faculty of Pharmaceutical Medicine	fpm@fpm.org.uk
Royal College of Physicians of Edinburgh	g.mcalister@rcpe.ac.uk
Royal College of Physicians of Ireland	No email
Royal College of Physicians of London	No email
Royal College of Physicians & Surgeons of Glasgow	No email
Royal College of Psychiatrists	reception@rcpsych.ac.uk
Faculty of Public Health	marijanacuric@fph.org.uk
Royal College of Radiologists	nquiries@rcr.ac.uk
Faculty of Sexual & Reproductive Health	No email
Royal College of Surgeons of Edinburgh	mail@rcsed.ac.uk
Royal College of Surgeons of England	communications@rcseng.ac.uk
Royal College of Surgeons of Ireland	communications@rcsi.ie
Faculty of Forensic and Legal Medicine	forensic.medicine@fflm.ac.uk

For peer review only - http://bmjopen.bmj.com/site/about/guidelines.xhtml

Specialty	
Acute Medicine	gg-uhb.CTMedicineScot@nhs.net<br>Ct1recruitment@jrcptb.org.uk<br>nationalrecruitment@nhslothian.scot.nhs.uk<br>st3recruitment@jrcptb.org.uk
Anaesthetics	edemployment.tayside@nhs.net<br>Medicalrecruitment.tayside@nhs.net<br>nationalrecruitment@wm.hee.nhs.uk
Cardiology	national.recruitment@nhslothian.scot.nhs.uk<br>St3recruitment@jrcptb.org.uk
Cardiothoracic surgery	nationalrecruitment@nes.scot.nhs.uk
Chemical Pathology ST1	nationalrecruitment@nes.scot.nhs.uk
Clinical Genetics	nationalrecruitment@nes.scot.nhs.uk<br>St3recruitment@jrcptb.org.uk
Clinical Pharmacology	nationalrecruitment@nes.scot.nhs.uk<br>St3recruitment@jrcptb.org.uk
Clinical Radiology	nationalrecruitment@nes.scot.nhs.uk
Core Anaesthesia	edeployment.tayside@nhs.net<br>nationalrecruitment@wm.hee.nhs.uk
Core Medicine	gg-uhb.CTMedicineScot@nhs.net<br>Ct1recruitment@jrcptb.org.uk
Core Psychiatry	nhsg.scotmtenquiries@nhs.net<br>Nwd.psychiatryrecruitment@nw.hee.nhs.uk
Core Surgical Training	nationalrecruitment@nes.scot.nhs.uk
Dermatology	nationalrecruitment@nes.scot.nhs.uk
Emergency Medicine	ACCSEMrecruitment@nes.scot.nhs.uk
Endocrine	st3recruitment@jrptb.org.uk
Gastroenterology	st3recruitment@jrcptb.org.uk
General Surgery	nationalrecruitment@nes.scot.nhs.uk
Genitourinary medicine	nationalrecruitment@nes.scot.nhs.uk
Geriatric medicine	st3recruitment@jrcptb.org.uk
General Practice	gpstp@nes.scot.nhs.uk
Haematology	st3recruitment@jrcptb.org.uk
Histopathology	nationalrecruitment@nes.scot.nhs.uk
Intensive care medicine	icm@wm.hee.nhs.uk
Medical oncology	nationalrecruitment@nes.scot.nhs.uk<br>st3recruitment@jrcptb.org.uk
Neurology	nationalrecruitment@nes.scot.nhs.uk<br>st3recruitment@jrcptb.org.uk
Neurosurgery	nationalrecruitment@nes.scot.nhs.uk
Obstetrics & Gynaecology	nationalrecruitment@nes.scot.nhs.uk<br>obsjobs@nw.hee.nhs.uk<br>obsandgynscotmt.recruitment@nhs.net
Ophthalmology	vana.fadda@rcophth.ac.uk
Otolaryngology	st3recruitment@jrptb.org.uk
Paediatrics	aa-uhb.clinicalsmtpaeds-crosshouse@nhs.net<br>paedjobs@rcpch.ac.uk
Paediatric cardiology	nationalrecruitment@nes.scot.nhs.uk
Public Health	nationalrecruitment@nes.scot.nhs.uk
Trauma & Orthopaedics	nhshighland.st1traumaorthopaedics@nhs.net
Sexual health	specialtyofficer@fsrh.org

For peer review only

UK & Ireland Trainees Survey for Doctors & Dentists in Training
Cochrane UK & Ireland Trainees Advisory Group - Trainee Survey Working Group

Introduction

Thank you for completing this short survey, which should take no longer than 5 minutes for you to complete. It will ask you to report the extent to which you feel you practice evidence-based medicine or dentistry. The results will guide the Cochrane UK Trainees Advisory Group to develop resources that support you and other trainees in medicine and dentistry to apply the evidence base to your clinical work.

For peer review only

UK & Ireland Trainees Survey for Doctors & Dentists in Training
Cochrane UK & Ireland Trainees Advisory Group - Trainee Survey Working Group

Demographics

1. Which region are you currently training in?

2. Which specialty are you currently training in?
(choose N/A if you are a foundation trainee)

	Doctors	Dentists
Specialty		

Other (please specify)

3. Which stage of training pathway are you currently undertaking?

	Doctors	Dentists
Training stage		

Other (please specify)

4. Are you currently, or have you previously been in an academic training programme?

- Yes, currently
- Yes, previously
- No, never

5. If you have any additional qualification(s), please choose all appropriate. (If not, skip to the next page)

	Clinical	Research	Management/ Leadership	Statistics	Teaching	Other
Bachelors	☐	☐	☐	☐	☐	☐
Postgraduate Certificate	☐	☐	☐	☐	☐	☐
Postgraduate Diploma	☐	☐	☐	☐	☐	☐
Masters	☐	☐	☐	☐	☐	☐
Doctorate	☐	☐	☐	☐	☐	☐

Other (please specify)

UK & Ireland Trainees Survey for Doctors & Dentists in Training
Cochrane UK & Ireland Trainees Advisory Group - Trainee Survey Working Group

1. How frequently in your clinical practice would you refer to published literature* in order to determine the evidence-base for a specific action or intervention?

* Eg. Clinical Knowledge Summaries, NICE guidelines, systematic reviews, individual papers

- ◯ Never
- ◯ Less than once a month
- ◯ More than once a month but not every week
- ◯ At least once a week
- ◯ On most days

2. Which of the following prevent you from more regularly using the evidence base in your clinical practice? (choose all applicable)

- ☐ Lack of relevant/ high quality evidence in my specialty
- ☐ Insufficient time to consult the literature
- ☐ Dfficult to access resources from my Trust
- ☐ Lack of confidence in directly basing my decisions on published findings
- ☐ I do not think evidence would change my clinical practice
- ☐ Difficulty in interpreting the statistics in the literature
- ☐ Tendency to follow the generally accepted clinical practice in my department
- ☐ Not relevant to me or my specialty (state which specialty in the box below)
- ☐ Other (please specify in the box below)
- ☐ None

Free text box

3. Which of the following motivate you to explore the evidence base to inform your clinical practice? (choose all applicable)

- ☐ Lack of a relevant guideline
- ☐ Poor quality guideline(s)
- ☐ Encouraged to by senior colleagues
- ☐ Routine practice in my specialty (state which specialty in the box below)
- ☐ Awareness of negative clinical outcome as a result of non-evidence based clinical practice
- ☐ Desire to better understand how clinical decisions are made
- ☐ I do not use the evidence base
- ☐ Other (please specify in the box below)

Free text box

4. Would you feel comfortable querying a colleague's management plan based on your reading of the evidence?

- ○ Yes - I would feel comfortable querying a colleague's management at all levels
- ○ Yes - but only if that colleague was more junior/ less experienced than me
- ○ No
- ○ Depends on the situation (please elaborate in the box below)

Comments

5. Which of the following sources of scientific evidence have you previously consulted to inform your clinical practice? (choose all applicable)

- [ ] None
- [ ] Published original research
- [ ] Narrative review articles
- [ ] Systematic reviews/ meta-analyses
- [ ] Conference abstracts
- [ ] NICE Clinical Knowledge Summary
- [ ] Local guidelines/ reports
- [ ] National guidelines
- [ ] Reports from a specialty-specific organisatoin

Other (please specify)

6. Have you previously consulted the Cochrane Library to seek evidence to solve a clinical problem?

- ( ) Yes
- ( ) No

7. How confident do you feel searching for evidence?

	1 (Not at all)	2	3	4	5 (Very confident)
Confidence level	( )	( )	( )	( )	( )

8. How confident are you in interpreting basic statistics (e.g. confidence interval, relative risk) when reading a journal article?

	1 (Not at all)	2	3	4	5 (Very confident)
Confidence level	( )	( )	( )	( )	( )

9. How would you use social media and the internet for your training/ education? (choose all applicable)

- [ ] Not be interested in using social media and the internet for this purpose
- [ ] I prefer offline materials (eg. offline courses, conference stands)
- [ ] I would be interested in reading an evidence-based practice Twitter feed that updated during the day
- [ ] I would use the Cochrane UK Facebook page
- [ ] I would use forums on the Cochrane UK website
- [ ] I would read a blog (eg. 'Evidently Cochrane')
- [ ] I would listen to a podcast
- [ ] I would be interested in receiving email updates on evidence-based practice from Cochrane UK for junior doctors/ dentists
- [ ] I would be interested in an online journal club to improve my critical appraisal skills and evidence-based practice
- [ ] Other (please specify)

10. Please leave any additional comments or your email address if you wish to be contacted to discuss survey-related topics. Thank you very much for your contribution to the survey.

Cochrane UK & Ireland Trainees Advisory Group - Trainees Survey Working Group

If you want to find out about opportuinties to get involved, please visit our website: uk.cochrane.org/trainees

For peer review only

BMJ Open

Motivators & Barriers to Engagement with Evidence Based Practice Amongst Medical & Dental Trainees from the United Kingdom & Republic of Ireland: a National Survey

Journal:	*BMJ Open*
Manuscript ID	bmjopen-2019-031809.R1
Article Type:	Original research
Date Submitted by the Author:	20-Aug-2019

Complete List of Authors:	Hong, Bosun; Birmingham Dental Hospital; University of Birmingham<br>O'Sullivan, Eoin; Renal Medicine<br>Henein, Christin; University of Newcastle upon Tyne; Moorfields Eye Hospital NHS Foundation Trust<br>Jones, Christopher; University of Leeds, ;

**Primary Subject Heading**:	Evidence based practice
Secondary Subject Heading:	Dentistry and oral medicine, Medical management
Keywords:	MEDICAL EDUCATION & TRAINING, Postgraduate Education, Evidence-based practice

SCHOLARONE™
Manuscripts

Motivators & Barriers to Engagement with Evidence Based Practice Amongst Medical & Dental Trainees from the United Kingdom & Republic of Ireland: a National Survey

Bosun Hong (bosunhong@gmail.com)[1,2,3], Eoin O'Sullivan (eoindosullivan@gmail.com)[1,4], Christin Henein (christin.henein@gmail.com)[1,5,6] & Christopher M. Jones (c.jones1@leeds.ac.uk)[1,7,8]

[1]Cochrane UK & Ireland Trainees Advisory Group; [2]Birmingham Dental Hospital, Birmingham Community Healthcare NHS Foundation Trust, UK; [3]School of Dentistry, College of Medical & Dental Sciences, University of Birmingham, UK; [4]MRC Centre for Inflammation Research, Queen's Medical Research Institute, University of Edinburgh, UK; [5]Institute of Genetic Medicine, Newcastle University, UK, [6]National Institute for Health Research Biomedical Research Centre at Moorfields Eye Hospital NHS Foundation Trust and UCL Institute of Ophthalmology, UK; [7]Faculty of Biological Sciences, University of Leeds, UK; [8]Radiotherapy Research Group, Leeds Cancer Centre, The Leeds Teaching Hospitals NHS Trust, UK.

Short title: UK & Ireland Medical & Dental Trainee Engagement with Evidence Based Practice

Keywords: Evidence-based practice, Evidence-based medicine, Postgraduate education

Word count: 4,320 words **Number of pages:** 19
Number of tables: 2 **Number of figures:** 4
Supplementary files: 2

Funding: This research received no specific grant support from the public, commercial or not-for-profit sectors. BH was supported for the duration of this work by a National Institute for Health Research (NIHR) Academic Clinical Fellowship (ACF) in Oral Surgery. EOS was supported for the duration of this work by Kidney Research UK. CH was supported for the duration of this work by a NIHR ACF in Ophthalmology. CMJ was supported for the duration of this work by a NIHR ACF in Clinical Oncology and a Wellcome Trust N4 Clinical Research Training Fellowship (203914/Z/16/Z).

Competing interests: The authors declare that they have no relevant conflicts of interest.

Data sharing statement: Deidentified participant data are available from Dr Christopher Jones (c.jones1@leeds.ac.uk) on request. Raw study data will be made available within two weeks following an appropriate request.

Address all correspondence and requests for reprints to:
Christopher M. Jones
Level 7 LIGHT Laboratories
Faculty of Biological Sciences
University of Leeds
Leeds
LS2 9JT, UK.

For peer review only - http://bmjopen.bmj.com/site/about/guidelines.xhtml

ABSTRACT

Objectives: To explore the extent to which doctors and dentists in training within the United Kingdom (UK) and Republic of Ireland (RoI) engage in and with evidence-based practice (EBP), and to identify motivators and barriers to them doing so.

Design: An observational, pre-piloted web-based survey developed by a trainee-led focus group.

Setting: The survey instrument was disseminated to doctors and dentists in training within the UK and RoI during June 2017 via social media and through Deanery, Royal College and specialty-specific mailing lists.

Participants: Data from 243 trainees were analysed; 188 doctors from 31 specialties and 55 dentists from nine specialties. Responses were received from trainees at all stages of postgraduate training though the overall response rate was low.

Primary & secondary outcome measures: The motivators and barriers to, and the extent of, trainee-engagement with EBP.

Results: Cronbach's α was 0.83. Most trainees (87.6% [n=148] of doctors and 75.1% [n=39] of dentists) consulted the evidence base at least monthly, whilst 23.1% [n=39 doctors, 12 dentists] of both specialties did so daily. The two most commonly cited barriers to engagement with EBP for both doctors and dentists respectively were insufficient time (57.6% [n=95] and 45.1% [n=23]) and a tendency to follow departmental practice (40.6% [n=67] and 45.1% [n=23]). Key motivators for EBP included curiosity, following the example set by senior colleagues and a desire to avoid harm. Most trainees reported high levels of confidence interpreting evidence yet for 26.8% [n=45] of doctors and 36.5% [n=19] of dentists, medical hierarchy would impede them querying a colleague's management plan based on their own reading of the evidence.

Conclusions: Time, accepted departmental practice and the behaviour of senior clinicians all highly impact on trainee engagement with EBP. Given the low response rate, the extent to which these data represent the overall population is unclear.

For peer review only - http://bmjopen.bmj.com/site/about/guidelines.xhtml

ARTICLE SUMMARY

Strengths & limitations of this study

1. National survey detailing medical and dental trainees' level of engagement with evidence-based practice.

2. Provides policy-makers, trainers and healthcare organisations with evidence for areas in which trainees' engagement with evidence-based practice can be encouraged and facilitated.

3. Reach extends across the UK and Republic of Ireland but results may not represent trainees' experiences in other countries.

4. Both dentists and doctors in training sampled but modest sample size may limit generalisability.

For peer review only

For peer review only - http://bmjopen.bmj.com/site/about/guidelines.xhtml

INTRODUCTION

Evidence-based practice (EBP) describes the integration of a patient's values and a clinician's expertise with the contemporary scientific evidence base.(1) The concept encourages the implementation of knowledge gained through systematic scientific discovery into clinical decisions along with psychological rationale, individual clinical experience and patient preference. In doing so, it reduces the potential impact of physician bias whilst empowering all clinicians, including those with less experience such as trainees, to make decisions informed by high-quality evidence.(2) There is significant evidence for the overall benefit of implementing EBP.(3-5)

Within the United Kingdom (UK) and the Republic of Ireland (RoI), EBP is firmly embedded within both medical and dental curricula. Reflecting this, the General Medical Council recommends that new doctors must be able to 'access and analyse reliable sources of current clinical evidence and guidance and have established methods for making sure their practice is consistent with these'.(6) Similarly, the General Dental Council stipulates that upon registration, dental trainees should be able to apply an evidence-based approach and utilise critical thinking skills.(7)

Despite these recommendations and the known tangible benefits of EBP to patient outcomes and care, it is recognised that clinical practice is not universally 'evidence based'.(3, 4, 8) The barriers to EBP have been studied previously and are numerous.(9-11) Commonly cited examples include inadequate knowledge or awareness of relevant evidence, a lack of individual motivation to engage with the evidence base and difficulty reconciling available evidence with a clinical question.(9)

The extent to which these barriers specifically apply to postgraduate medical and dental trainees, and the degree to which they therefore engage with EBP, is for the most part unknown. Much of the evidence cited to date is instead drawn from grouped populations of undergraduate and postgraduate trainees, or from cohorts consisting of both trainees and more senior clinicians who are beyond completion of training.(10, 12) The characteristics of the working environment, clinical responsibilities and drivers for learning of postgraduate trainees may differ from those of non-training clinicians or undergraduates. Hence, an understanding of the role the postgraduate training environment plays in moulding trainees' desire and ability to engage with the evidence base is

For peer review only - http://bmjopen.bmj.com/site/about/guidelines.xhtml

important. It may, for instance, guide regulators and educators alike in designing interventions to promote the translation of EBP teaching into clinical work for this population. It may also provide training programme coordinators and senior clinicians with an insight into how best to support their more junior colleagues to engage in EBP.

The Cochrane UK & Ireland Trainees Advisory Group was established as the trainee-led arm of Cochrane UK & Ireland in 2016. It is formed of 15 medical and dental trainees from across the UK & RoI who represent a diverse range of specialties and who are together tasked with enhancing trainee-engagement with EBP. Given the lack of evidence concerning trainees' engagement with EBP in the contemporary UK and RoI healthcare systems, the Trainees Advisory Group undertook a national survey focused on trainees' use of the evidence base in routine clinical practice and the wider motivators for and barriers to use of the evidence base amongst these doctors and dentists in training.

METHODS

Study design

We conducted a cross-sectional study targeting doctors and dentists in postgraduate training from across the UK and RoI using an anonymous electronic survey between June and October 2017.

Survey instrument

Members of the Cochrane UK & Ireland Trainees Advisory Group formed a focus group in order to develop an online survey specifically for this study. The rationale for designing a bespoke instrument for this survey was based on our literature review which revealed no validated instrument to cover all areas of focus for our survey. Drawing on their own experiences, the Trainees Advisory Group defined the following as areas of focus for the survey: (a) trainee engagement with EBP, (b) motivators and barriers to engagement with EBP; and (c) trainees' preferences towards approaches to enhance engagement in EBP. Engagement in this context was defined as maintaining sufficient knowledge of the contemporary evidence base relevant to one's clinical role to influence views of or specific practice within that role.

For peer review only - http://bmjopen.bmj.com/site/about/guidelines.xhtml

The survey instrument was devised and refined through an iterative process that included two pilot surveys with 19 postgraduate trainees who represented 13 specialties in the fields of medicine and dentistry, and seven geographical locations within the UK and RoI. In its final form (**Supp. File: Web-Based Survey Instrument**) the survey instrument consisted of 14 closed-ended questions, five of which also permitted optional free-text response and two of which incorporated a five-point Likert scale. Included questions focussed on the aforementioned areas of interest defined by the focus group. Demographic data were also collected and included each trainee's specialty and training stage, prior academic training and their location, subdivided as either RoI or by UK deanery. We collected data using a cloud-based online survey programme (SurveyMonkey, California, USA).

Study population & survey dissemination

Trainees in a recognised UK or RoI training post within any medical or dental specialty formed the target population for this study. Full registration with the General Medical Council was not required in order to ensure that responses from Foundation Year 1 doctors were considered. UK and RoI trainees are widely geographically distributed and are overseen by a large number of professional organisations, including regional deaneries and various Royal Colleges. There is no single point of contact for either doctors or dentists in training. In order to enhance coverage of trainees eligible to participate in this survey, we used a phased approach to its dissemination that over a three-week period incorporated both a social media arm and direct approaches to trainees via email and online training portfolios (see **Fig. 1**).

In week one (commencing 30/06/2017), we disseminated the survey via email to relevant professional organisations with oversight of doctors and dentists in training across the UK and RoI. We also used the email marketing service MailChimp® to disseminate the survey to medical deaneries across the UK and to 49 trainees subscribed to the Cochrane UK & Ireland Trainees Advisory Group newsletter (see **Supp. Table 1**). MailChimp® enabled us to check whether our emails were opened by the recipient organisations but could not facilitate tracking of the actual survey response rate as the number of trainees receiving the survey link, including via other modalities such as social media, was not known. We also distributed to UK dental deaneries following

For peer review only - http://bmjopen.bmj.com/site/about/guidelines.xhtml

For peer review only

approval from the UK Committee of Postgraduate Dental Deans and Directors (COPDEND). In both instances, administrators within these professional bodies were asked to distribute the survey to trainees overseen by their organisation via direct email or through use of the NHS training ePortfolio, to which all medical trainees have access.

During this same week, a link to the survey was posted online using the Cochrane UK Twitter account (@CochraneUK), which at the time had in excess of 40,000 followers. In line with an *a priori* planned social media strategy, this link was re-tweeted nine times by this same account and by members of the Cochrane UK & Ireland Trainees Advisory Group within the same week. Tweets were flagged for the attention of the Twitter accounts of relevant Royal Colleges and other bodies overseeing trainees.

In week two, we once again contacted those professional bodies who had been contacted during week one but who had not responded to the first survey approach. Contemporaneously, further Tweets outlining the survey were distributed by the Cochrane UK & Ireland Twitter account. These continued into the third week. The survey subsequently remained open until October 2017 in order to permit further responses to be collected.

Data analysis

The unit of analysis of this study is the individual respondent (trainee). Data are provided for doctors and dentists separately where possible. There were insufficient participant numbers to analyse by specialty. Data are shown for separate stages of training where numbers allow. Descriptive analyses were undertaken using a statistical software, R (R Core Team, version 3.4.3, 2013). We computed the frequency of answers and where relevant expressed this as a percentage of total respondents. For Likert-type scaled response answers, we assumed answers to be an interval-level measurement to allow visualisation using bar plots.

We conducted a thematic analysis of the free text responses using an inductive approach, following the phases of thematic analysis outlined by Braun and Clarke.(13) Having become familiarised with the data, we identified initial codes using a qualitative data analysis software (NVivo, QSR International Pty Ltd., version 12, 2018) and organised the codes into potential themes. The identified themes were reviewed and refined before final

For peer review only - http://bmjopen.bmj.com/site/about/guidelines.xhtml

categorisation. In addition, we have searched for and retained deviant responses that do not fit into the themes but nevertheless make a valuable contribution to the overall message of this work.

Ethical approval

There was no requirement for specific ethical approval for this study. The survey was distributed to medical and dental trainees across the UK and RoI. Trainees received the survey via an email or eportfolio link, or may have followed a link to the survey via social media. In all instances neither we nor any other person or professional organisation collected or had access to any identifiable data relating to trainees completing the survey. Indeed, all trainees were reminded at the outset of survey completion that they were under no obligation to complete the questionnaire and that their details and answers would remain anonymous. All participants were informed of the study's purpose and content, and all completed the survey on a device of their choosing to which the researchers had no access and all were free to stop participating at any point. Under NHS Health Research Authority guidelines, NHS Research Ethics Committee (REC) approval is not required for research of this kind in England, Scotland, Wales and Northern Ireland (as per the HRA Decision Toolkit: http://www.hra-decisiontools.org.uk/ethics/). Similarly, under Health Service Executive guidelines, research of this nature does not require REC approval in the Republic of Ireland (as per https://www.hse.ie/eng/services/list/5/publichealth/publichealthdepts/research/rec.html).In addition, there was no incentive offered for survey completion.

Patient & public involvement

There was no direct patient involvement in the design or conduct of this study.

RESULTS

Internal consistency

The survey had high internal consistency with a Cronbach's α of 0.83.

Respondent demographics

For peer review only

For peer review only - http://bmjopen.bmj.com/site/about/guidelines.xhtml

A total of 243 responses were received; 188 (83.3%) from doctors in 31 specialties, and 55 (16.7%) from dentists in 9 specialties (see **Table 1**). Most (53% [n=123]) trainees were specialty training 3 (ST3) level or above in seniority, and the majority (70% [n=166]) had not previously undertaken formal postgraduate academic training. Responses were received from trainees in RoI and in 20 out of 21 UK deaneries. The geographical distribution of respondents is summarised in **Table 2.**

Engagement with EBP

To assess trainees' level of engagement with EBP, those responding to the survey were asked how frequently they would refer to published literature to determine the evidence base for a specific action or intervention (**Fig. 2a**). The majority of doctors (87.6% [n=148]) and dentists (75.1% [n=39]) reported consulting the evidence base at least monthly. An identical proportion (23.1% [n=39 doctors and 12 dentists]) of the two professions consulted the evidence base daily.

Trainees were additionally asked to report which sources of evidence they had previously consulted in order to inform their practice (**Fig. 2b**). For both doctors and dentists, national (95.8% [n=160] and 88.5% [n=46] respectively) and local (81.4% [n=136] and 67.3% [n=35]) guidance, NICE Clinical Knowledge Summaries (82.6% [n=138] and 86.5% [n=45]), systematic reviews (85.6% [n=143] and 84.6% [n=44]) and published original research (79.6% [n=133] and 63.5% [n=33]) formed the principal sources of information.

Trainees' self-ratings of their confidence in searching evidence and interpreting basic statistics were strongly negatively skewed (**Figs. 3 a** and **b)**, though this distribution became normal when trainees with prior academic experience were removed from the analysis. As highlighted by **Figs. 3**, confidence in these key skills also increases with training stage.

When asked for their perception of social media and the internet for education to support their EBP, the majority of respondents (72.0% [n=157]) were supportive of its use. Popular forms of engagement were via email updates (53.7% [n=117]), an online journal club (47.3% [n=101]) and through a podcast (40.4% [n=88]).

For peer review only - http://bmjopen.bmj.com/site/about/guidelines.xhtml

Motivators for EBP

There was some variation between doctors and dentists in the factor respondents highlighted as their motivation for consulting the evidence base (**Fig. 4a**). For dentists, the most frequently cited reason was to better understand how clinical decisions are made (84.3% [n=43]), a curiosity noted by 54.6% (n=90) of responding doctors. Conversely, whilst 42.4% (n=123) of doctors reported that consulting the evidence base was routine practice in their specialty, this was the case for just 17.6% [n=9] of dentists. For both doctors and dentists, lack of a relevant guideline (37.0% [n=61] and 39.2% [n=20] respectively), encouragement from senior colleagues (37.0% [n=61] and 45.1% [n=23]) and an awareness of the potential for a negative clinical outcome from not consulting the evidence base (34.6% [n=57] and 41.2% [n=21]) were other frequently reported motivators for engagement in EBP.

These findings were supported by thematic analysis of free-text comments, which revealed that motivators for EBP included trainees' desire to provide a good standard of care at either the individual patient level, or for public health trainees, at the population level. The responses captured the inspiration to do so, which included examples of harm caused to patients by non-EBP, involvement in research projects, and following the example set by senior colleagues:

> "I think it is important to review literature as often ingrained practice has no real proof of benefit. I also think that by understanding the evidence you learn better and you understand its applicability and are better able to decide if it applicable for your patient cohort."

> "The ethical implications of continuing to practice in an evidence-light environment following the withdrawal of the Liverpool Care Pathway"

> "…My consultants are also very much into [EBP] & quote papers all the time. I feel that I'm expected to know the evidence behind the treatments I offer patients & I think this is a good thing as it makes me much more confident at recommending a particular treatment to a patient."

For peer review only - http://bmjopen.bmj.com/site/about/guidelines.xhtml

Barriers to engagement with EBP

Time, access & local practices

Barriers to EBP reported by trainees are summarised in **Fig. 4b**. The two most commonly cited barriers for doctors and dentists respectively were insufficient time (57.6% [n=95] and 45.1% [n=23]) and a tendency to follow departmental practice (40.6% [n=67] and 45.1% [n=23]). Limited access to relevant evidence from the workplace was also commonly reported as a barrier, though more frequently amongst doctors (40.6% [n=67]) than dentists (25.5% [n=13]).

Thematic analysis of free-text responses supported these data, and highlighted that trainees considered time and access as important barriers to EBP:

> "By far the greatest barrier to taking an evidence-based approach to my work is the lack of access to the evidence. I am a public health registrar with a passion for the wider determinants of health. At the moment I have very limited Athens access through the training scheme, but following this I'm not sure if I will even have that…"

> "I am limited by the insufficient electronic journal access provided as part of NHS Athens, which is inferior to the access provided by a good university account. I do manage to work around this, but it takes extra time and limits the sources I can use"

Some trainees felt that the access issue was two-fold; electronic access to articles being the first hurdle and having the information technology facility within the NHS premises to allow access to the information being the next one:

> "…the evidence base is spread across multiple resources, some of which are, with the best will in the world difficult to use in day to day practice, from crappy, locked down NHS computers. If they are free to use at all."

For peer review only

For peer review only - http://bmjopen.bmj.com/site/about/guidelines.xhtml

"Poor wifi signal often frustrates me when I want to look at a resource such as NICE or UptoDate on the wards"

Some trainees felt that the use of evidence was not prioritised in postgraduate training despite its importance, and that those who require further training on this may struggle to find the time due to other priorities:

"We are not taught how to use the evidence base. Yeah sure, the local library might offer some introductory courses (if you are in a good hospital) but it is unacceptable that 'moving & handling' is a mandatory course but searching and using the evidence base is treated as optional."

"I was lucky enough to learn about evidence-based research at medical school and taught how to conduct literature reviews but it is something I just don't have the time for now."

Limited evidence

Dentists in training more commonly cited a lack of relevant or high-quality evidence as a barrier than doctors (37.3% [n=19] vs 12.7% [n=21]). Thematic analysis also revealed the challenges trainees face in specialties in which evidence is scarce and where studies have unclearly reported methodology or questionable generalisability to their patients:

"…there is a really lack of randomised controlled trials in my specialty and rejection of them by some of the large research groups. The question of how much evidence is enough evidence in palliative medicine remains to be answered."

Reliance on secondary evidence such as local, specialty-specific, or national guidelines was evident in the free-text responses. Trainees perceived secondary evidence to be of high quality and therefore their default first choice of avenue for evidence:

For peer review only - http://bmjopen.bmj.com/site/about/guidelines.xhtml

"I use individual papers very rarely. Think Royal College of Obstetrics and Gynaecology does a good job of synthesising current information and producing guidance on common topics."

"commonly used guidelines have been memorised – e.g. asthma, transient loss of consciousness"

Hierarchy

When trainees were asked if they would feel comfortable querying a colleague's management plan based on the trainee's reading of the evidence, 57.7% [n=97] of doctors and 51.9% [n=27] of dentists were unconditionally so. Other trainees reported that they would feel confident only if the colleague was more junior or less experienced than them (19.6% [n=33] and 30.8% [n=16]), or that this depended on the situation (15.5% [n=26] and 11.5% [n=6]). A smaller proportion of respondents stated that they would feel uncomfortable about querying a colleague's management in any case (7.1% [n=12] and 5.8% [n=3]).

Upon analysing the free-text responses, a passive attitude of following generally accepted departmental practice was a frequent feature. It was evident that the working relationship with, or the personality of a senior colleague was more important to trainees than the seniority itself when considering whether to question a senior colleague's decision. Embedded within the responses was a situational analysis process that trainees go through in their mind before deciding to approach their senior colleague. Some trainees have developed a strategy where their decision to question a senior colleague would be conditional – for example, only if the impact to the patient is large, only if they predict a positive response from the colleague, only if they have direct responsibility for care (i.e. "asked to complete the treatment"):

"Some colleague's personalities may colour my comfort level to query/discuss management!"

"Consultants who are confident and progressive would be willing to discuss. Some seniors would find this threatening as they are insecure in their own practice."

For peer review only - http://bmjopen.bmj.com/site/about/guidelines.xhtml

"…you have to read the situation…"

"It really depends on so many factors (e.g. who is the manager, who are you, how long you have been there, what type of relation you have...) In general, I would only do it if I feel that there will be a positive response. If I feel that it is only going to generate conflict, then, what's the point?"

"… I pick my fights as such and only raise it if I think it is important/going to make a difference etc."

EBM malaise

There was also a response which pointed out that a clinician's unfounded preconceived notions may act as a barrier to a scientific enquiry:

"The problem is that the concept of "evidence" has lost its meaning. People use for all sorts of things...I mean that people, when faced by a public health issue, they don't ask themselves "What does the evidence say about how to resolve this issue?". What usually happens is that they have a pre-defined position and they ask themselves "What is the evidence that supports my position?" That influences how searches are done, how bias is assessed and so on."

DISCUSSION

The extent to which doctors and dentists in contemporary training schemes engage with the evidence base, and the barriers and motivators to them doing so, is underexplored. We outline here a nationwide survey of UK and RoI doctors and dentists from a large number of specialties and at all stages of training. Most actively engage with literature at least monthly, whilst a quarter does so daily. Dentists in training most frequently cited curiosity as their main motivator for EBP and reported a lack of relevant evidence as a key barrier to further engagement with the evidence base. On the other hand, doctors frequently cited their engagement with EBP in the context of accepted practice for the departments in which they work. For both doctors and dentists, a lack of time and poor access to relevant literature were key barriers to further engagement in EBP. Trainees nevertheless reported

For peer review only - http://bmjopen.bmj.com/site/about/guidelines.xhtml

For peer review only

high levels of confidence in searching for evidence and interpreting basic statistics. However, the majority also reported that they would not query a more senior colleague based on their own reading of the evidence, thereby potentially limiting the extent to which their confidence translates in to EBP.

The notion that trainees appreciate EBP but do not consider their practice to be consistently evidence-based is supported elsewhere in the literature.(14-16) For instance, a survey of pre-registration house officers (currently termed foundation trainees in the UK) in the Mersey Deanery revealed that trainees considered critical appraisal skills to be relevant to their practice yet only just over half felt that their clinical practice was based on the best available evidence.(14) Similarly, a survey of obstetrics and gynaecology trainees in West Midlands revealed that whilst most trainees perceived EBP as a positive concept, relatively few consistently referred to literature.(15) It is of interest that many of the barriers identified in these more limited specialty-specific study are reflected in the broad trainee population reported here.

Our findings have a number of additional implications, which the Cochrane UK & Ireland Trainees Advisory Group have reviewed with a view to formulating strategic, concrete plan to promote EBP. The higher reported confidence in EBP of trainees who have undertaken formal postgraduate academic training demonstrates the value of academic training schemes to healthcare settings. Notably, that the hierarchy and departmental practice so considerably shape the degree to which trainees engage in EBP demonstrates the parallel importance of ensuring an appropriate training environment. In view of this, the Trainees Advisory Group plans to collect examples of good practice as a result of postgraduate academic training and positive influence from colleagues or department practice, and disseminate them through its online platforms. The data depicted in our survey additionally demonstrate a clear need to support busy trainees in digesting evidence, whether through directly managing their workload or by ensuring that sources of evidence are readily available and their conclusions efficiently consumable. Other implications of this work are likely to be harder to address. The paucity of relevant evidence highlighted by dental trainees is one such area but may be challenged by promoting greater awareness of EBP within the profession.

Limitations

For peer review only - http://bmjopen.bmj.com/site/about/guidelines.xhtml

The number of trainees reached by the survey is not known. However, there are over 60,000 doctors and nearly 3,000 dentists in training in the UK alone.(14-15) The overall response rate was therefore low and the data are prone to responder bias. Responses were however captured from a large number of specialties and good geographical coverage was achieved. The degree to which the data depicted here represent the overall population is, therefore, unclear and the outcomes of this survey require validation in a larger cohort. Further, a number of specialties were not represented or were but by only a small number of respondents, and other specialties such as public health were relatively over-represented. As such, it is impossible to characterise differences in the data between specialties and training stages. The use of email and social media to contact doctors and dentists also provides the potential for selection bias. The extent to which these results are applicable to healthcare settings outside of the UK and RoI is in addition unclear.

This survey nevertheless had a wide geographical reach across both the UK and RoI, incorporated a significant number of specialties and features responses from a large number of doctors and dentists in training. Internal consistency was also high, as measured by a Cronbach's α of 0.83. The data provide a number of clear themes, some of which are specific to the postgraduate trainee population. For example, the important influence of hierarchy, both as a motivator and a barrier, is a trainee-specific theme that emerged prominently as a result of our focus on the postgraduate trainee population. These themes provide a starting point for further research and intervention from both healthcare professionals and policymakers. The validity of these themes is supported by their concordance with historical evidence (16-18), and their relevance is reflected from the post-modernising medical careers cohort from which they are derived.

CONCLUSION

Time and a lack of access to key resources limit the ability of doctors and dentists in training within the UK and RoI to engage with EBP. Whilst the majority of these trainees report high levels of confidence in searching and interpreting statistics in the literature, a significant proportion highlight that hierarchy and departmental practice significantly shape their EBP. The extent to which these data vary between specialties and by stage of training is unknown and requires further research. Healthcare professionals and policymakers should ensure that trainees

For peer review only - http://bmjopen.bmj.com/site/about/guidelines.xhtml

are provided with the time and ability to efficiently access sources of evidence, and should engender cultures in which EBP is encouraged.

AUTHOR CONTRIBUTIONS

BH and CMJ devised, piloted and carried out the survey, and collated data. BH prepared the first draft of the manuscript. BH, EOS, CH and CMJ led the analysis of data. All authors contributed to the interpretation of results and revisions of the draft manuscript. All authors have read and approved the final version of the manuscript.

ACKNOWLEDGEMENTS

We are grateful to trainees for taking the time to respond to this survey and to the various professional bodies who distributed the survey. We are also grateful for support from Cochrane UK, which is funded by the National Institute for Health Research (NIHR), and to Dr Emma Plugge for her critical appraisal of the manuscript.

For peer review only

For peer review only - http://bmjopen.bmj.com/site/about/guidelines.xhtml

REFERENCES

1. Sackett DL, Rosenberg WM, Gray JA, Haynes RB, Richardson WS. Evidence based medicine: what it is and what it isn't. 1996. Clin Orthop Relat Res. 2007;455:3-5.
2. Lewis SJ, Orland BI. The importance and impact of evidence-based medicine. J Manag Care Pharm. 2004;10(5 Suppl A):S3-5.
3. Lenfant C. Shattuck lecture--clinical research to clinical practice--lost in translation? N Engl J Med. 2003;349(9):868-74.
4. Awtry EH, Loscalzo J. Aspirin. Circulation. 2000;101(10):1206-18.
5. Stafford RS, Radley DC. The underutilization of cardiac medications of proven benefit, 1990 to 2002. J Am Coll Cardiol. 2003;41(1):56-61.
6. The General Medical Council. Outcomes for graduates. London, UK; 2018
7. The General Dental Council. Preparing for practice: dental team learning outcomes for registration. 2012.
8. O'Connor GT, Quinton HB, Traven ND, Ramunno LD, Dodds TA, Marciniak TA, et al. Geographic variation in the treatment of acute myocardial infarction: the Cooperative Cardiovascular Project. JAMA. 1999;281(7):627-33.
9. Cabana MD, Rand CS, Powe NR, Wu AW, Wilson MH, Abboud PA, et al. Why don't physicians follow clinical practice guidelines? A framework for improvement. JAMA. 1999;282(15):1458-65.
10. Taylor R, Reeves B, Ewings P, Binns S, Keast J, Mears R. A systematic review of the effectiveness of critical appraisal skills training for clinicians. Med Educ. 2000;34(2):120-5.
11. Hannes K, Norre D, Goedhuys J, Naert I, Aertgeerts B. Obstacles to implementing evidence-based dentistry: a focus group-based study. J Dent Educ. 2008;72(6):736-44.
12. Sullivan KJ, Wayne C, Patey AM, Nasr A. Barriers and facilitators to the implementation of evidence-based practice by pediatric surgeons. J Pediatr Surg. 2017;52(10):1666-73.
13. Clarke V, Braun V, Delgadillo J. Using thematic analysis in counselling and psychotherapy research: A critical reflection. Couns Psychother Res 2018; 18: 107-110.
14. General Medical Concil. The State of medical education and practice in the UK. 2017. Available online: https://www.gmc-uk.org/static/documents/content/SoMEP-2017-final-full.pdf. Accessed 19th August 2019.
15. COPDEND. Dental Recruitment Applicant Handbook. 2019. Available online: https://www.copdend.org/wp-content/uploads/2019/01/DentalRecruitmentApplicantHandbook2019.pdf. Accessed 19th August 2019.
16. Doran T, Maudsley G, Zakhour H. Time to think? Questionnaire survey of pre-registration house officers' experiences of critical appraisal in the Mersey Deanery. Med Educ. 2007;41(5):487-94.
17. Awonuga AO DL, Khan KS, Taylor R. Postgraduate obstetrics and gynaecology trainees' views and understanding of evidence-based medicine. Med Teach. 2000;22:27-9.

For peer review only - http://bmjopen.bmj.com/site/about/guidelines.xhtml

18. Manikam L HA, Fosker H, Wong MHY, Banerjee J, Lakhanpaul M, Knight A, Littlejohns P. What drives junior doctors to use clinical practice guidelies? A national cross-sectional survey of foundation doctors in Engalnd & Wales. BMC Med Educ. 2015;15:227.

For peer review only

For peer review only - http://bmjopen.bmj.com/site/about/guidelines.xhtml

FIGURE LEGENDS

Figure 1: A flowchart depicting the survey dissemination approach, including via social media.

Figure 2: Trainee engagement with evidence based practice, including **(a)** the frequency with which trainees consult published literature in order to evaluate a clinical problem and **(b)** the sources trainees would typically consult in order to assess the evidence base.

Figure 3: Perceived confidence in searching evidence **(a)** and interpreting basic statistics **(b)** on a scale of 1 (least confident) to 5 (most confident).

Figure 4: A summary of reported **(a)** motivators and **(b)** barriers to engagement with EBP.

For peer review only

For peer review only - http://bmjopen.bmj.com/site/about/guidelines.xhtml

TABLE 1: Survey respondents by profession and specialty.

	**Doctors** (n=188)		**Dentists** (n=55)	
	N	%	N	%
**Specialty**				
Acute internal medicine	1	0.5		
Anaesthetics	7	3.7		
Cardiology	1	0.5		
Chemical pathology	1	0.5		
Clinical radiology	2	1.0		
Community sexual and reproductive health	12	6.4		
Dermatology	2	1.1		
Emergency medicine	5	2.7		
Foundation training	24	12.8	27	49.1
General medicine	5	2.7		
General practice	6	3.2	1	1.8
General psychiatry	2	1.1		
General surgery	6	3.2		
Geriatric medicine	4	2.1		
Haematology	1	0.5		
Oral histopathology			1	1.8
Intensive care medicine	1	0.5		
Infectious diseases	2	1.1		
Neurosurgery	1	0.5		
Obstetrics & gynaecology	20	10.6		
Occupational medicine	2	1.1		
Ophthalmology	2	1.1		
Oral and maxillofacial surgery*	1	0.5	3	5.5
Oral medicine			8	14.5
Oral surgery			5	9.1
Orthodontics			2	3.6
Paediatric and perinatal pathology	1	0.5		
Paediatric dentistry			4	7.3
Paediatric medicine	9	4.8		
Palliative medicine	2	1.1		
Periodontology as a monospecialty			1	1.8
Plastic surgery	1	0.5		
Public health medicine	57	30.3		
Renal medicine	3	1.6		
Respiratory medicine	2	1.1		
Restorative dentistry			2	3.6
Special care dentistry			1	1.8
Urology	1	0.5		
Vascular surgery	1	0.5		
Unknown	3	1.6		

*Oral and maxillofacial surgery is recognised as a specialty by the General Medical Council and as such their specialty trainees are registrants of the General Medical Council. In addition to specialty training, the specialty facilitates dental core training and therefore the majority of their junior staff are dentists

For peer review only

For peer review only - http://bmjopen.bmj.com/site/about/guidelines.xhtml

TABLE 2: Survey respondents by profession, location of training and training stage.

	**Doctors** (n=188)		**Dentists** (n=55)	
	N	%	N	%
**Respondent's location of training**				
Scotland	13	6.9	25	45.5
East Midlands, England	9	4.8	6	10.9
East of England	5	2.7	3	5.5
London, England	33	17.6	2	3.6
North East, England	3	1.6	1	1.8
North West, England	10	5.3	4	7.3
South East, England	22	11.7	2	3.6
South West, England	17	9.0	2	3.6
West Midlands, England	15	8.0	6	10.9
Yorkshire and the Humber, England	19	10.1	1	1.8
Wales	4	2.1	0	0.0
Northern Ireland	3	1.6	0	0.0
Republic of Ireland	28	14.9	2	3.6
Unknown	7	3.7	1	1.8
**Training stage**				
Foundation years	22	11.7	28	50.9
Core/Specialty trainee 1/2	39	20.7	15	27.3
Specialty trainee 3	30	16.0	4	7.3
Specialty trainee 4	31	16.4	2	3.6
Specialty trainee 5	20	10.6	2	3.6
Specialty trainee 6	9	4.8	1	1.8
Specialty trainee 7	8	4.3	0	0.0
Specialty trainee 8	0	0.0	2	3.6
RoI – Intern	3	1.6	0	0.0
RoI – Senior House Officer	4	2.1	1	1.8
RoI – Specialty Registrar	10	5.3	0	0.0
Unknown	12	6.4	0	0.0

For peer review only - http://bmjopen.bmj.com/site/about/guidelines.xhtml

SUPPLEMENTARY MATERIAL

Supplementary Table 1: A summary of professional organisations contacted via email

Supplementary File: Web-based survey instrument.

For peer review only

For peer review only - http://bmjopen.bmj.com/site/about/guidelines.xhtml

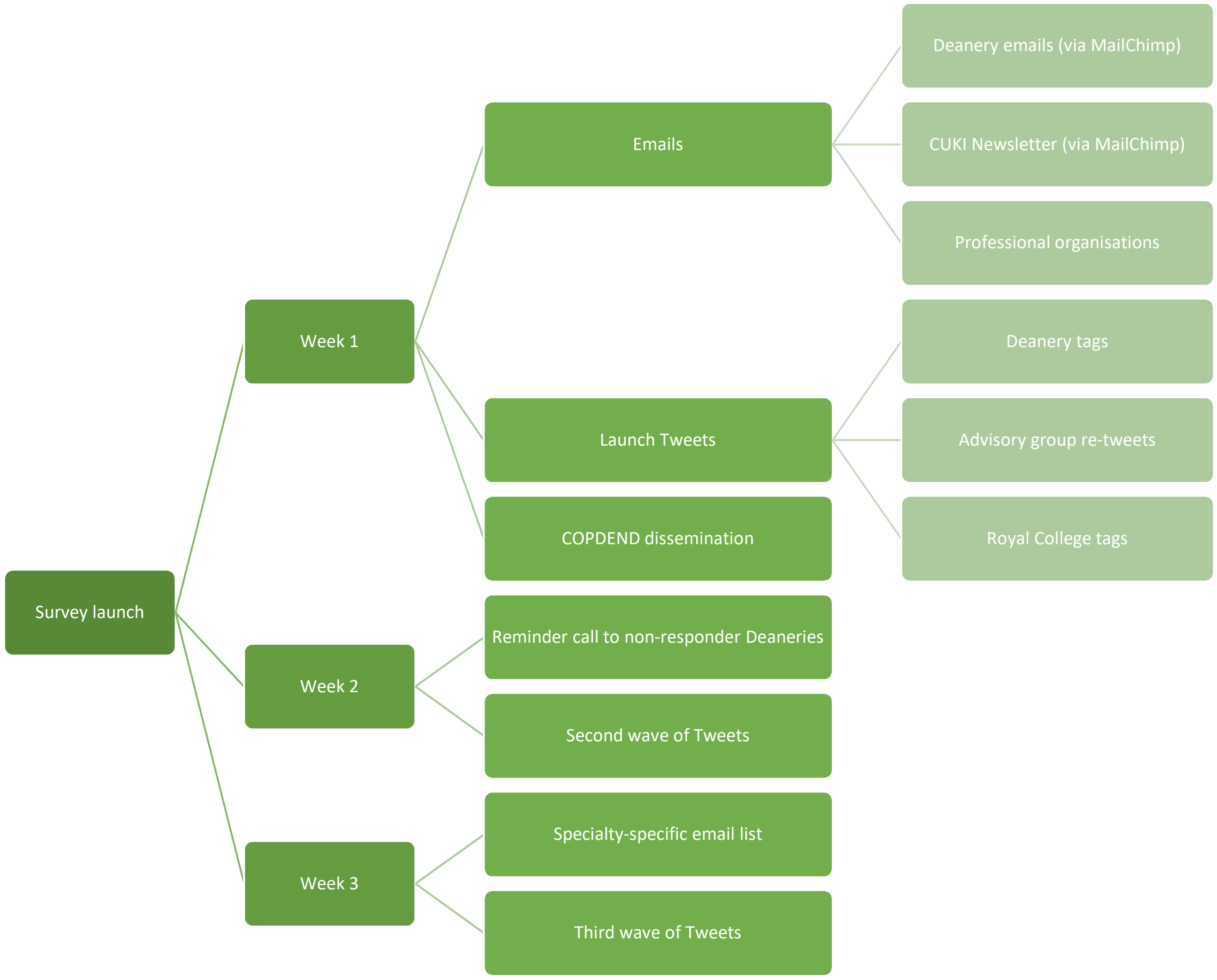
Survey launch
Week 1
Week 2
Week 3
Emails
Launch Tweets
COPDEND dissemination
Reminder call to non-responder Deaneries
Second wave of Tweets
Specialty-specific email list
Third wave of Tweets
Deanery emails (via MailChimp)
CUKI Newsletter (via MailChimp)
Professional organisations
Deanery tags
Advisory group re-tweets
Royal College tags

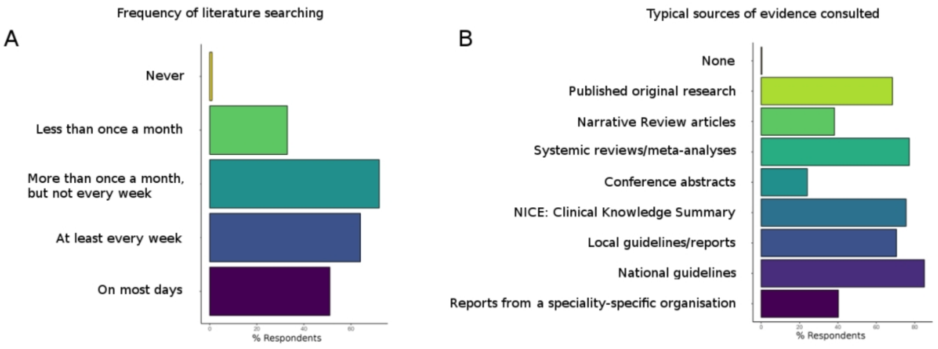

Figure 2

120x47mm (300 x 300 DPI)

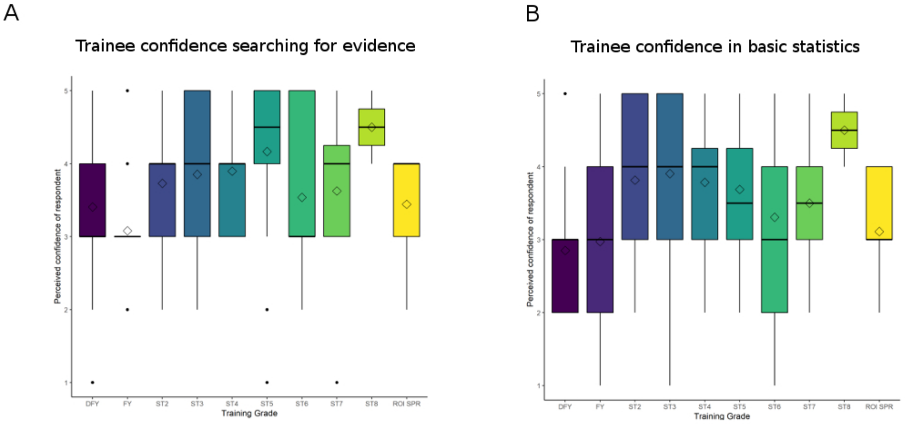

Figure 3

93x44mm (300 x 300 DPI)

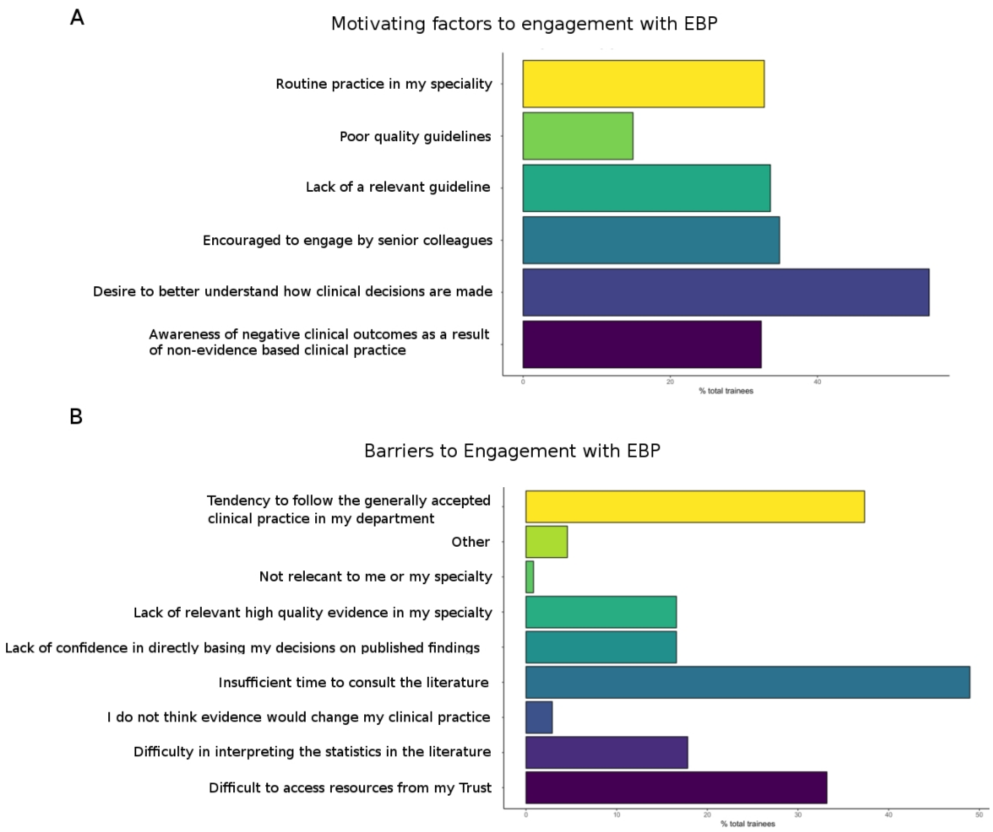

Figure 4

112x100mm (300 x 300 DPI)

Motivators & Barriers to Engagement with Evidence Based Practice Amongst Medical & Dental Trainees from the United Kingdom & Republic of Ireland: a National Survey

Bosun Hong, Eoin O'Sullivan, Christin Henein, Christopher M. Jones

Supplementary Table 1. A summary of professional organisations contacted via email

Organisation / Specialty	Contact
**Deanery**	
All	hee.pressoffice@nhs.net
Health Education West Midlands	specialtyrecruitment@wm.hee.nhs.uk
Health Education East Midlands	communications.em@hee.nhs.uk
Health Education East of England	midlandsandeast.comms@nhs.net
Health Education North East	Enquiries.NE@hee.nhs.uk
Health Education North West	info.nw@hee.nhs.uk
Health Education Yorkshire & The Humber	contactus.yh@hee.nhs.uk
Health Education Thames Valley	enquiries.TV@hee.nhs.uk
Health Education Wessex	Reception.wx@hee.nhs.uk
Health Education South West	info.SW@hee.nhs.uk
Health Education South London	info.sl@hee.nhs.uk
Health Education North West London	info.nwl@hee.nhs.uk
Health Education North Central & East London	info.ncel@hee.nhs.uk
Health Education Kent, Surrey & Sussex	rotations.lase@hee.nhs.uk
Wales	walesdeanery@cardiff.ac.uk
**Royal College**	
Royal College of Anaesthetists	info@rcoa.ac.uk
Royal College of Emergency Medicine	No email
Royal College of General Practitioners	membership@rcgp.org.uk
Faculty of Intensive Care Medicine	ficm@rcoa.ac.uk
Royal College of Obstetricians & Gynaecologists	pressoffice@rcog.org.uk
Faculty of Occupational Medicine	Leigh.Harrison@FOM.ac.uk
Royal College of Paediatrics & Child Health	enquiries@rcpch.ac.uk
Royal College of Pathologists	info@rcpath.org
Faculty of Pharmaceutical Medicine	fpm@fpm.org.uk
Royal College of Physicians of Edinburgh	g.mcalister@rcpe.ac.uk
Royal College of Physicians of Ireland	No email
Royal College of Physicians of London	No email
Royal College of Physicians & Surgeons of Glasgow	No email
Royal College of Psychiatrists	reception@rcpsych.ac.uk
Faculty of Public Health	marijanacuric@fph.org.uk
Royal College of Radiologists	nquiries@rcr.ac.uk
Faculty of Sexual & Reproductive Health	No email
Royal College of Surgeons of Edinburgh	mail@rcsed.ac.uk
Royal College of Surgeons of England	communications@rcseng.ac.uk
Royal College of Surgeons of Ireland	communications@rcsi.ie
Faculty of Forensic and Legal Medicine	forensic.medicine@fflm.ac.uk

For peer review only - http://bmjopen.bmj.com/site/about/guidelines.xhtml

Specialty	
Acute Medicine	gg-uhb.CTMedicineScot@nhs.net<br>Ct1recruitment@jrcptb.org.uk<br>nationalrecruitment@nhslothian.scot.nhs.uk<br>st3recruitment@jrcptb.org.uk
Anaesthetics	edemployment.tayside@nhs.net<br>Medicalrecruitment.tayside@nhs.net<br>nationalrecruitment@wm.hee.nhs.uk
Cardiology	national.recruitment@nhslothian.scot.nhs.uk<br>St3recruitment@jrcptb.org.uk
Cardiothoracic surgery	nationalrecruitment@nes.scot.nhs.uk
Chemical Pathology ST1	nationalrecruitment@nes.scot.nhs.uk
Clinical Genetics	nationalrecruitment@nes.scot.nhs.uk<br>St3recruitment@jrcptb.org.uk
Clinical Pharmacology	nationalrecruitment@nes.scot.nhs.uk<br>St3recruitment@jrcptb.org.uk
Clinical Radiology	nationalrecruitment@nes.scot.nhs.uk
Core Anaesthesia	edeployment.tayside@nhs.net<br>nationalrecruitment@wm.hee.nhs.uk
Core Medicine	gg-uhb.CTMedicineScot@nhs.net<br>Ct1recruitment@jrcptb.org.uk
Core Psychiatry	nhsg.scotmtenquiries@nhs.net<br>Nwd.psychiatryrecruitment@nw.hee.nhs.uk
Core Surgical Training	nationalrecruitment@nes.scot.nhs.uk
Dermatology	nationalrecruitment@nes.scot.nhs.uk
Emergency Medicine	ACCSEMrecruitment@nes.scot.nhs.uk
Endocrine	st3recruitment@jrptb.org.uk
Gastroenterology	st3recruitment@jrcptb.org.uk
General Surgery	nationalrecruitment@nes.scot.nhs.uk
Genitourinary medicine	nationalrecruitment@nes.scot.nhs.uk
Geriatric medicine	st3recruitment@jrcptb.org.uk
General Practice	gpstp@nes.scot.nhs.uk
Haematology	st3recruitment@jrcptb.org.uk
Histopathology	nationalrecruitment@nes.scot.nhs.uk
Intensive care medicine	icm@wm.hee.nhs.uk
Medical oncology	nationalrecruitment@nes.scot.nhs.uk<br>st3recruitment@jrcptb.org.uk
Neurology	nationalrecruitment@nes.scot.nhs.uk<br>st3recruitment@jrcptb.org.uk
Neurosurgery	nationalrecruitment@nes.scot.nhs.uk
Obstetrics & Gynaecology	nationalrecruitment@nes.scot.nhs.uk<br>obsjobs@nw.hee.nhs.uk<br>obsandgynscotmt.recruitment@nhs.net
Ophthalmology	vana.fadda@rcophth.ac.uk
Otolaryngology	st3recruitment@jrptb.org.uk
Paediatrics	aa-uhb.clinicalsmtpaeds-crosshouse@nhs.net<br>paedjobs@rcpch.ac.uk
Paediatric cardiology	nationalrecruitment@nes.scot.nhs.uk
Public Health	nationalrecruitment@nes.scot.nhs.uk
Trauma & Orthopaedics	nhshighland.st1traumaorthopaedics@nhs.net
Sexual health	specialtyofficer@fsrh.org

For peer review only - http://bmjopen.bmj.com/site/about/guidelines.xhtml

UK & Ireland Trainees Survey for Doctors & Dentists in Training

Cochrane UK & Ireland Trainees Advisory Group - Trainee Survey Working Group

Introduction

Thank you for completing this short survey, which should take no longer than 5 minutes for you to complete. It will ask you to report the extent to which you feel you practice evidence-based medicine or dentistry. The results will guide the Cochrane UK Trainees Advisory Group to develop resources that support you and other trainees in medicine and dentistry to apply the evidence base to your clinical work.

For peer review only

UK & Ireland Trainees Survey for Doctors & Dentists in Training
Cochrane UK & Ireland Trainees Advisory Group - Trainee Survey Working Group

Demographics

1. Which region are you currently training in?

2. Which specialty are you currently training in?
(choose N/A if you are a foundation trainee)

	Doctors	Dentists
Specialty		

Other (please specify)

3. Which stage of training pathway are you currently undertaking?

	Doctors	Dentists
Training stage		

Other (please specify)

4. Are you currently, or have you previously been in an academic training programme?

- Yes, currently
- Yes, previously
- No, never

5. If you have any additional qualification(s), please choose all appropriate. (If not, skip to the next page)

	Clinical	Research	Management/ Leadership	Statistics	Teaching	Other
Bachelors	☐	☐	☐	☐	☐	☐
Postgraduate Certificate	☐	☐	☐	☐	☐	☐
Postgraduate Diploma	☐	☐	☐	☐	☐	☐
Masters	☐	☐	☐	☐	☐	☐
Doctorate	☐	☐	☐	☐	☐	☐

Other (please specify)

UK & Ireland Trainees Survey for Doctors & Dentists in Training
Cochrane UK & Ireland Trainees Advisory Group - Trainee Survey Working Group

1. How frequently in your clinical practice would you refer to published literature* in order to determine the evidence-base for a specific action or intervention?

* Eg. Clinical Knowledge Summaries, NICE guidelines, systematic reviews, individual papers

- ○ Never
- ○ Less than once a month
- ○ More than once a month but not every week
- ○ At least once a week
- ○ On most days

2. Which of the following prevent you from more regularly using the evidence base in your clinical practice? (choose all applicable)

- ☐ Lack of relevant/ high quality evidence in my specialty
- ☐ Insufficient time to consult the literature
- ☐ Dfficult to access resources from my Trust
- ☐ Lack of confidence in directly basing my decisions on published findings
- ☐ I do not think evidence would change my clinical practice
- ☐ Difficulty in interpreting the statistics in the literature
- ☐ Tendency to follow the generally accepted clinical practice in my department
- ☐ Not relevant to me or my specialty (state which specialty in the box below)
- ☐ Other (please specify in the box below)
- ☐ None

Free text box

3. Which of the following motivate you to explore the evidence base to inform your clinical practice? (choose all applicable)

- ☐ Lack of a relevant guideline
- ☐ Poor quality guideline(s)
- ☐ Encouraged to by senior colleagues
- ☐ Routine practice in my specialty (state which specialty in the box below)
- ☐ Awareness of negative clinical outcome as a result of non-evidence based clinical practice
- ☐ Desire to better understand how clinical decisions are made
- ☐ I do not use the evidence base
- ☐ Other (please specify in the box below)

Free text box

4. Would you feel comfortable querying a colleague's management plan based on your reading of the evidence?

- ○ Yes - I would feel comfortable querying a colleague's management at all levels
- ○ Yes - but only if that colleague was more junior/ less experienced than me
- ○ No
- ○ Depends on the situation (please elaborate in the box below)

Comments

5. Which of the following sources of scientific evidence have you previously consulted to inform your clinical practice? (choose all applicable)

- ☐ None
- ☐ Published original research
- ☐ Narrative review articles
- ☐ Systematic reviews/ meta-analyses
- ☐ Conference abstracts
- ☐ NICE Clinical Knowledge Summary
- ☐ Local guidelines/ reports
- ☐ National guidelines
- ☐ Reports from a specialty-specific organisatoin

Other (please specify)

6. Have you previously consulted the Cochrane Library to seek evidence to solve a clinical problem?

- ○ Yes
- ○ No

7. How confident do you feel searching for evidence?

	1 (Not at all)	2	3	4	5 (Very confident)
Confidence level	○	○	○	○	○

8. How confident are you in interpreting basic statistics (e.g. confidence interval, relative risk) when reading a journal article?

	1 (Not at all)	2	3	4	5 (Very confident)
Confidence level	○	○	○	○	○

9. How would you use social media and the internet for your training/ education? (choose all applicable)

- [ ] Not be interested in using social media and the internet for this purpose
- [ ] I prefer offline materials (eg. offline courses, conference stands)
- [ ] I would be interested in reading an evidence-based practice Twitter feed that updated during the day
- [ ] I would use the Cochrane UK Facebook page
- [ ] I would use forums on the Cochrane UK website
- [ ] I would read a blog (eg. 'Evidently Cochrane')
- [ ] I would listen to a podcast
- [ ] I would be interested in receiving email updates on evidence-based practice from Cochrane UK for junior doctors/ dentists
- [ ] I would be interested in an online journal club to improve my critical appraisal skills and evidence-based practice
- [ ] Other (please specify)

10. Please leave any additional comments or your email address if you wish to be contacted to discuss survey-related topics. Thank you very much for your contribution to the survey.

Cochrane UK & Ireland Trainees Advisory Group - Trainees Survey Working Group

If you want to find out about opportuinties to get involved, please visit our website: uk.cochrane.org/trainees

y only
